# Enhancement of healthful novel sugar contents in genetically engineered sugarcane juice integrated with molecularly characterized *ThSyGII (CEMB-SIG2)*

**DOI:** 10.1038/s41598-022-23130-y

**Published:** 2022-11-03

**Authors:** Mudassar Fareed Awan, Sajed Ali, Muhammad Shahzad Iqbal, Muhammad Nauman Sharif, Qurban Ali, Idrees Ahmad Nasir

**Affiliations:** 1 Department of Biotechnology, Knowledge Unit of Science, University of Management and Technology Sialkot Campus, Lahore, Pakistan; 2 Centre of Excellence in Molecular Biology, University of the Punjab, Lahore, Pakistan; 3 Department of Biochemistry, University of Okara, Okara, Pakistan; 4 Department of Plant Breeding and Genetics, Faculty of Agricultural Sciences, University of the Punjab, Lahore, Pakistan; 5 Four Brothers Genetics, Lahore, Pakistan

**Keywords:** Biotechnology, Plant biotechnology

## Abstract

Enhancement of sugar contents and yielding healthful sugar products from sugarcane demand high profile scientific strategies. Previous efforts to foster manipulation in metabolic pathways or triggering sugar production through combating abiotic stresses fail to yield high sugar recovery in *Saccharum officinarum* L. Novel sucrose isomers trehalulose (TH) and isomaltulose (IM) are naturally manufactured in microbial sources. In pursuance of novel scientific methodology, codon optimized sucrose isomerase gene, *Trehalulose synthase gene II(CEMB-SIG2)* cloned under dual combined stem specific constitutive promoters in *pCAMBIA1301* expression vector integrated with Vacuole targeted signal peptide (VTS) to concentrate gene product into the vacuole. The resultant mRNA expression obtained by Real Time PCR validated extremely increased transgene expression in sugarcane culms than leaf tissues. Overall sugar estimation from transgenic sugarcane lines was executed through refractometer. HPLC based quantifications of Trehalulose (TH) alongside different internodes of transgenic sugarcane confirmed the enhancement of boosted sugar concentrations in mature sugarcane culms. *Trehalulose synthase gene II* receptive sugarcane lines indicated the unprecedented impressions of duly combined constitutive stem regulated promoters. Transgenic sugarcane lines produce highest sugar recovery percentages, 14.9% as compared to control lines (8.5%). The increased sugar recovery percentage in transgenic sugarcane validated the utmost performance and expression of *ThSyGII* gene .High Profile Liquid chromatography based sugar contents estimation of Trehalulose (TH) and Isomaltulose (IM) yielded unprecedented improvement in the whole sugar recovery percentage as compared to control lines.⁠

## Introduction

Manipulation of metabolic pathways in sugarcane (*Saccharum officinarum* L.) adds a promising approach towards synthesizing highly valuable compound in this crop^1,2^. Various methodologies of genetic engineering led to production and manipulation of beneficial biomolecules^3^. Sugarcane by-products accomplished immense attention owing to their increased demand and applicability in food industries. Their usage as feedstock, bio-energy input and raw material in various food processing units becomes rampant^4^. Sucrose (SUC), an abundant disaccharide accompanied with non-reducing characteristics is harvested and extracted from sugarcane juice as a primary food material. It is also employed as transporting machinery for carbohydrates invascular bundles of plants^5^. Being a product of glycosidic linkage between glucose (GLU) and fructose (FRU), SUC molecule naturally contains a large number of isomers. These isomers have identical functionalities but exhibit different structural features^6^. Trehalulose (TH) and Isomaltulose (IM) havegreat demand as natural sweeteners and are also exploited in modern food industrieson wide scale.These are the permanent parts of nutritious food items like honey, jam and jellies^7^. Microbial conversion of SUC to its isomers is an intrinsic characteristic pertaining to copious microbial community. This isomeric property helps these microbes in achieving superiority from other competing microbial species^8^. Different enzymes including sucrose isomerase (SI), IM synthase and TH synthase play pivotal role in sequestering SUC to its advantageous alternatives like IM and TH^9^. Diverse biochemical methods are administered to yield precious products from genetically engineered protein expression vectors^10^. Sucrose isomers have unique features, lacking in original SUC molecule including increased acid stability, acariogenicity and lower glycemic index^11^. Slow digestibility of IM and TH makes them ideal candidates for diabetic patients. The abrupt escalation of blood sugar levels can be reduced remarkably hence proved healthful for diabetic patients^12^. Although all sucrose isomers are attractive candidates with multiple benefits but TH exhibits unique feature of enhanced level of solubility than exists in SUC^13^. Literature witnessed that SI presented a viable strategy by converting highly dissolvable SUC to TH added benefits for consumers^14^. These SI enzymes are considered very costly and exist only in microbes making unreachable for common usage^15^. Moreover, in addition to other benefits, SI enzymes need no cofactor for their optimum efficiency and also perform in multistep pathway withlow free energy^14,16^.The reported SI belongs to same TIM-barrel family having thirteen molecules derived from glycosyltransferases. The SI enzymes vary significantly in mode of action, energy requirements, kinetic conversion rates, ratio of the product obtained and range of IM or TH produced as the result of their actions at particular conditions^17^. Microbial strains, *Pentoea dispers a* isolate UQ68J^18^ reported to manufacture highest level of IM, 91% while *Pseudomonas mesoacidophilia*^19^ exhibited high proficiency in causing boosted ratios of TH than IM from SUC. Some unconfirmed studies from whitefly, *Bemisia argentifolii* were also reported^20^. Many microbial sucrose isomerase genes (SIGs) were isolated, sequenced and reported to databases^21^. Codon optimization and gene characterization have opened new windows to retrieve SIGs from original sources, synthesize artificially, clone and transformation in target plant genotypes.

Integration of target SIGs in *pCAMBIA1301* under the combined dual promoters (pUbi-CmYMV) to yield maximum conversion of SUC to IM and TH, is considered a novel promising approach. In plants isomers metabolise slowly therefore highly efficient supply of isomers is made possible via metabolic source in cytoplasm from juvenile growing tissues^22^. Foregoing scientific studies focus on attaining boosted sugar production by the efficient conversion of IM from SUC which proved successful. The potential of TH has not been exploited properly in the past. This sugar isomer was highly neglected and its potential remain un-addressed by food biotechnologists^23^. Multiple bacterial strains have biochemical machinery for synthesizing SI enzymes and yield TH in culms frequently^24^.

Current research work investigated the vibrant abstruse activity of modified *Trehalulose synthase gene II(ThSyGII)* inside sugarcane cytosolic and vacoular region. The combinatorial expression performance of single and dual promoters, polyubiquitine (pUbi) and cestrum yellow mosaic virus (CmYMV) were also evaluated. Real Time PCR was exploited to quantify mRNA expression in leaf and stem tissues of transgenic lines. The research work also observed brix and high performance liquid chromatography (HPLC) based quantification results of sugar contents to calculate sugar recovery percentage (SRP) against control non-transgenic lines. The present study is essential in enhancing the income of sugarcane producers. Enhancement in SRP will directly increase capacity of growers ultimately boosts their economic profile. Moreover, this study also provided opportunities regarding harvesting healthful sugar contents including TH and IM with lower digestion rate, less glycemic index and easily digestibility^25^. The introduction and generation of an efficient TH containing sugarcane juice with additional benefits was the ultimate aim of this work. Molecular and biochemical approaches were coordinated to uphold a multidiscipline research innovative approach.

## Material and methods

### Molecular construction of plant expression vector

**Figure 1:**
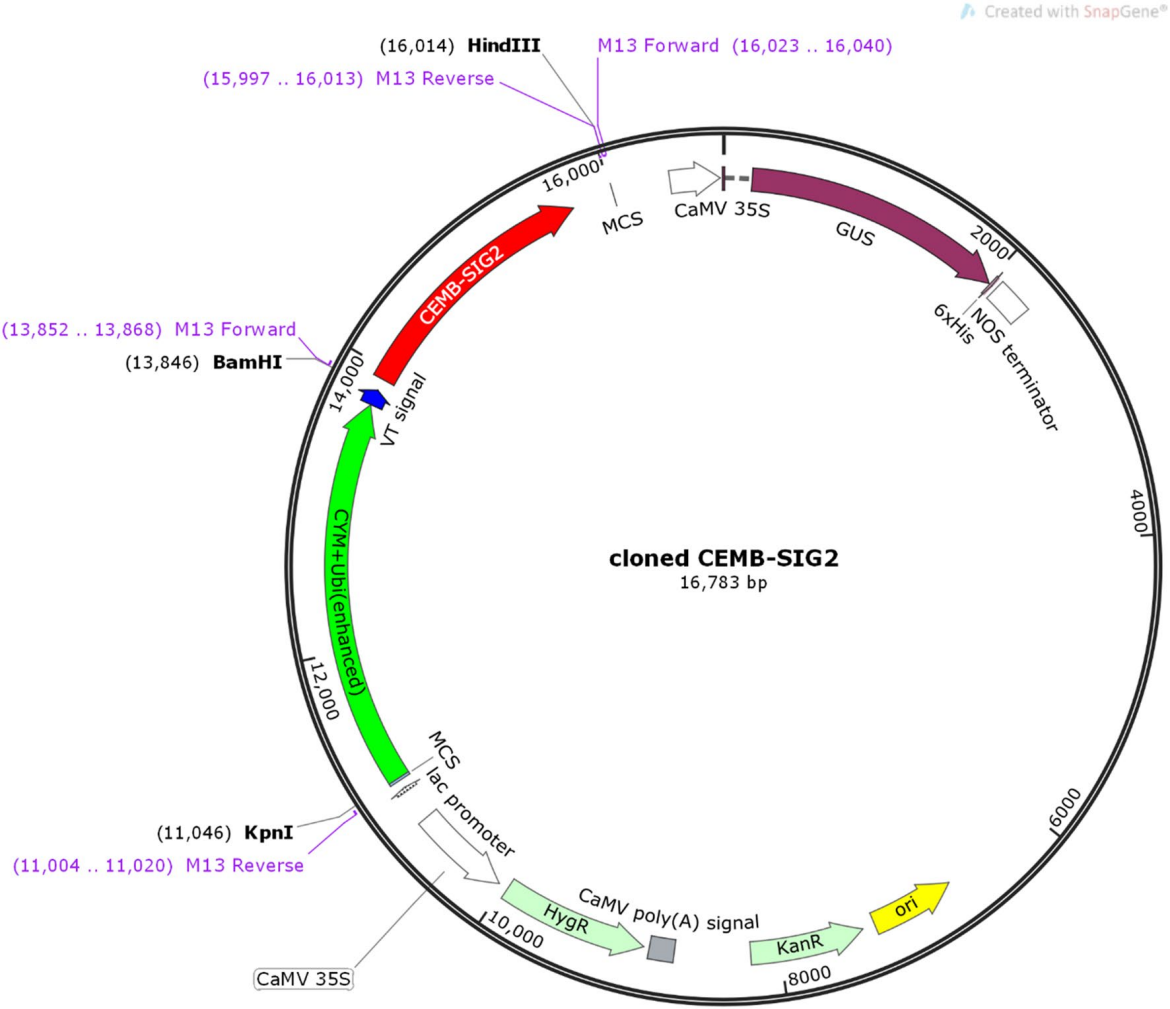
Circular gene map (16,783 bp) exhibiting inclusion of cloned *ThSyGII(CEMB-SIG2)* in red arrow driven CmYMV and pUbi promoters (green arrow) while VTS was shown in blue colour.

Reported sequence from NCBI database was retrieved and subjected to codon optimization in accordance with sugarcane (*Saccharum officinarum* L.). The synthesis was obtained from Integrated DNA Technology (IDT) Private Ltd. The codon optimized modified *ThSyGII* with 1755 bp encodes 584 amino acid long protein which was used as SI precursor. The similarity index of codon optimized sequence was determined by running N-BLAST analysis, P-BLAST was also executed to validate 100% synthesis of target protein. Concentrated synthetic gene *ThSyGII* (4 mg) was collected in pUC57 vector, synthetic construct was diluted (40 µg) accordingly forexperimental use. Transgene *ThSyGII* driven under the combined influence of two promoters including pUbi and CmYMV, leader sequences terminated by nopaline synthase gene (Nos). The vacuole targeted sequence (VTS) and endoplasmic reticulum leader sequence (ELS) were also integrated in this gene assembly to govern gene product inside specific stem vacuole. The complete gene construct consists of 2195 bp sequence. Restriction endonuclease *KpnI* was added at upstream while *HindIII* was integrated at downstream, the end of the terminator sequence. The complete gene cassette (2180 bp) (Fig. 1) was cloned in plant expression vector, pCAMBIA1301 screened by kanamycin (Kan^R^), hygromycin (Hyg^R^) and tetracycline (Tet^R^) resistant genes. Gene construct was excised from ampicillin selected bacterial expression vector, pUC57, and ligated to new destination in pCAMBIA1301 between *KpnI* and *HindIII* sites. The linear and circular map of gene construct was indicated in Fig. 1.

### Particle bombardment of genetic construct to sugarcane explant

The cloned gene construct was purified and subjected to transformation in sugarcane explants. Superior sugarcane line HSF-246 were collected from Ayyub Agriculture Research Institute Faisalabad (AARI) and grown at glass house facilities available in Centre of Excellence in Molecular Biology (CEMB) Lahore. Mature leaf whorls from topical portion was cut into small cylindrical dimensions and subjected to callus induction media as reported in past studies^26^. Healthy sugarcane calli plates were stored under dark conditions to prevent pre-mature growth. Highly concentrated gene construct containing *ThSyGII* (5 µg) was purified andadsorbed to sterilized washed tungsten particles by slow centrifugation process. The adsorption of highly concentrated gene construct with integrated *ThSyGII* was bombarded by particle gene gun method as described^20^. An optimum distance between gene gun and target calli plates was adjusted, 26 mmHg vacuum pressure was upheld inside the chamber and DNA adsorbed tungsten particles were bombarded to target tissues under helium controlled 110 pounds/inch^2^ pressure. Bombarded and controlled calli plates were incubated at dark for 48 h so that recovery could be made. Bombarded putative transgenic calli were shifted to zero Murashiage and Skoog (MS) media.

### Antibiotic screening and plant growth conditions

All putative transgenic sugarcane plants grown from calli plates were shifted to glass test tubes for initial screening against hygromycin (100 mg/mL) drug as selection marker. Initially screened and surviving plants were shifted into pots for further growth. Putative transgenic plants were placed under glass house conditions accomplished with 16 h light period and 8 h dark conditions. Temperature was calibrated at 28 ± 2°C and watering was done twice a day to juvenile putative transgenic plants. After one month, plants were acclimatized at nursery in open environment and carefulhandling was executed according to guidelines.

### PCR confirmationof putative transgenic calli

Three months old putative transgenic, antibiotic resistant screened sugarcane plants were further subjected to PCR amplifications. Fresh sugarcane leaves were collected in liquid nitrogen bucket for DNA extraction purpose. Purification of DNA from putative transgenic leaf samples was executed following protocols described in DNA purification kit (catalog #NA2110). Putative transgenic plants survived under drug selection media (MS Media supplemented with 100 µg/ml hygromycin drug)were subjected to PCR amplifications so that integration of *ThSyGII* might be confirmed. Primer sequences were orchestrated through Primer3 (https://primer3.ut.ee/*)* online tool. The following primer sequences were retrieved and employed for PCR amplifications. Forward: 5`-GGTTGGATAAAGGGGTCTCT-3`, Reverse: 5`-AGGGATAGTTCGTCATTCCA-3`. The product size for this PCR confirmation was set at 543 bp.

### Real time estimation of stem and leaf expression levels of *ThSyGII* in transgenic lines

Fresh leaf and stem samples were collected from PCR positive transgenic sugarcane lines. The RNA extraction was carried out by employing TRIZOL method. Tissues were ground by mortar and pestle followed by addition of TRIZOL (1 mL). The purified extracted RNA from leaf and stem tissues was collected separately on 1.5 mL eppendorf tubes, washed properly by absolute ethanol. Synthetic You-Prime beads were employed for executing reverse transcription and subsequent PCR amplifications using Taq polymerase (Promega) according to instructions given in the manufacturer’s manual. The 35 cycle protocol was adopted following; denaturation (95°C for 45 s), annealing (58°C for 30 s) and extension (72°C for 1 min) to complete DNA amplification. The primer sequences exploited in Real Time PCR were *SIGII-F-*5’GTTCTCCGCTACCTCCTACC 3’ and *SIGII-R-*5’ACCTGATAGAAGACGGCCTG 3’*.*

### Extraction of cane juice for brix & polarity percentages

PCR positive, *ThSyGII* harbored transgenic sugarcane lines along with control plants were sampled in triplicate for sugar estimation analysis. Sugarcane juice was extracted from stalks and brix test was administered to calculate commercial cane sugar (CCS) also known as SRP. After spectral acquisition, different sections of internodes were crushed for extraction of cane juice. Squeezing from various parts of the internode was executed to produce a real representative sample. Internodal wrapping of plastic bag was necessary to avoid cross contamination. For brix measurement, 20 ml of cane juice from *ThSyGII* expressing sugarcane line and 200 ml of sample cane juice were required for polarity (pol%) percentage calculation. Only brix values were employed for calibration purpose. Refractometer was employed to measure brix from transgenic cane juice samples. Polarimeter was administered to calculate *pol%* which was further exploited to find CCS commonly called as SRP. Extraction of cane juice and estimation of brixwas executed according to protocol^27^.

### Quantifications of TH via HPLC in different transgenic internodal stalks

Fresh samples were obtained from selected transgenic lines, targeted from mid-point of internodes. The radial sections were selected and considered representative of the whole sugarcane line. Smaller stalk samples were weighed (about 0.20 g FW), put onto the 1.5 mL microcentrifuge tube, frozen in liquid nitrogen, incubated on ice and vigorous centrifugation was done at the speed of 14000* g* continuously for10 min at 4 °C, juice drops went down to eppendorf tube and were readyfor further analysis. Sampled cane juice was boiled for approximately 4 min to inactivate all enzymes. Centrifugation was executed at 16000 g for 18 min, time and again to remove insoluble materials. This whole cane extraction process yielded quantity of sugar which stood equal to sugar obtained through conventional crushing procedure. Close record of fresh weights before and after juice extraction was kept carefully. The temperature requirements for sugarcane tissues stood at 75°C while for juices 90°C. Water contents from sample tissues were also evaluated carefully for measurement purpose. The protocol adopted for HPLC was administered according to scientific studies reported previously^28^. FRU, SUC, GLU, IM and TH were quantified after achieving iscocratic HPLC, done at increased pH level adjusted by alkaline compounds (130 mm NaOH). The HPLC (Dionex BioLC system, Sunnyvale, CA) was run with PA20 analytical anion exchange column and quad waveform pulsed ED. The calibration was also executed against dilution series of IM and TH standards for every sample batch. Appropriate dilution mechanism was adopted by adjustment of cane concentrations fixing SUC molecule as standard in cane juice. Sugar estimation profile for genetically engineered sugarcane lines was developed at both wet and dry forms. The relative sugar contents(60%) in internode tissues were also extracted after centrifugation process. Moreover nearly 10% reduction of juice to solute ratio was also included relative to the first expressed juice according to industry estimation mechanism.

### Statistical analysis of field grown transgenic sugarcane morphological datasets

Sugarcane samples from transgenic and non-transgenic control sugarcane plants were subjected to data collection for their morphological characters. Seven morphological traits including plant height, leaf length, internodal length, girth, no. of tillers, leaf area index and number of internodes were calculated according to protocol adopted previously^29^. Sugar estimation by SRP was already calculated by following recommended reported brix protocol^30^.

## Results and discussion

### Cloning confirmation of *ThSyGII (CEMB-SIG2)* in plant expression vector

**Figure 2:**
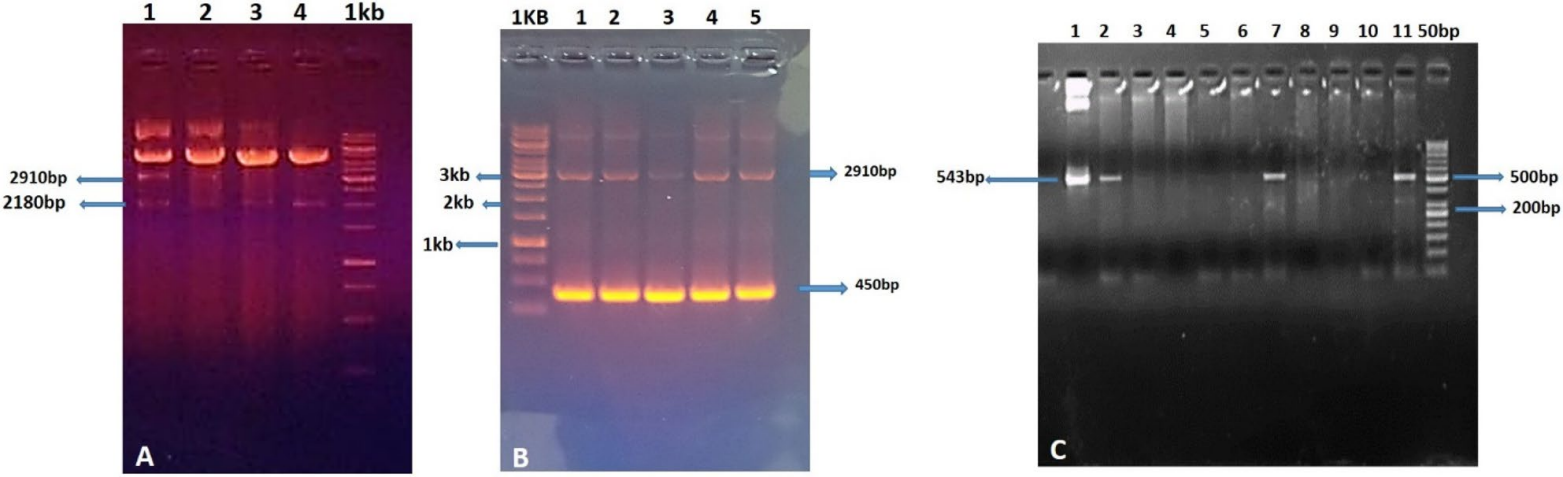
(**A**) Restriction digestion picture demonstrates excision and show confirmations of dual promoters (CmYMV-Ubi), 2910 bp cut with *KpnI* & *BamHI* (lane 4) and *ThSyGII (CEMB-SIG2)*2180 bp, cut with *BamHI* & *HindIII* (lane1, 2 & 3), lane 5 shows undigested plant expression vector while lane 1 shows 1 Kb ladder (**B**). Restriction digestion of promoters from bacterial cloning vector cut with *KpnI* and *BamH1*. (**C**) PCR amplifications of *ThSyGII* trigerred by sequence specific primers showing amplicon (543 bp) in lanes 1, 2, 7 and 11 while L lane indicates 1 kb ladder. Complete original uncropped gel pictures are provided in supplementary information file as fig. S-2A, S-2B and S-2C.

Integration and inclusion of synthetic gene construct was confirmed by restriction digestion analysis and PCR amplifications (Fig. 2A). Fragment size 2910 bp in Fig. 2A cut with specific restriction endonucleases (*KpnI and BamH1*), fragment 2180 bp for *ThSyGII*cut with *BamH1* and *HindIII* confirmed cloning in plant expression vector while Fig. 2B showed integration of dual promoters in bacterial vector. Figure 2C shows PCR amplification results of 543 bp fragment describing successful integration of *ThSyGII* gene in sugarcane genome.

### In-vitro phenotypic expression of *ThSyGII(CEMB-SIG2)* bombarded putative sugarcane calli

**Figure 3:**
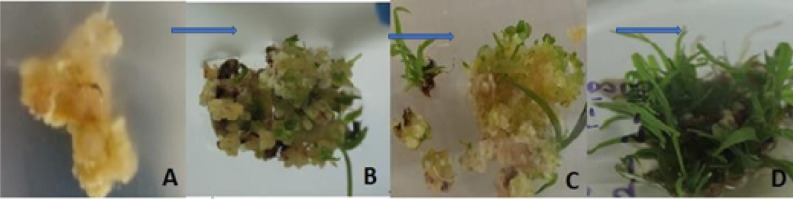
(**A**) Putative *ThSyGII* embedded calli shifted to MS media plates nourished with auxins and cytokinins (**B**) Calli initiated regeneration after 7 days of 28 °C 16 h light and 8 h dark conditions (**C**) Regeneration of putative transgenic calli after 14 days (**D**) Shoots appeared with capacity after 30 days of media nourishments.All full length original uncropped figures are provided in the supplementary information file as figure S-3A, S-3B, S-3C and S-3D.

Immediately after particle gene bombardment experiment intopetri plate 2(PP2) with gene construct *ThSyGII (CEMB-SIG2)*, calli were shifted to MS plates enriched with auxin and cytokinin. The Fig. 3A,B showed different regeneration events starting from day 1 to day 30. The phenotypes of *ThSyGII* intruded calli after bombardment with particle gene gun were shown in Fig. 3C,D. Different phases of development from calli to full regenerated transgenic plants can be visualized.

### Screening of PCR amplified putative transgenic sugarcane plants harboring *ThSyGII (CEMB-SIG2)*

**Figure 4:**
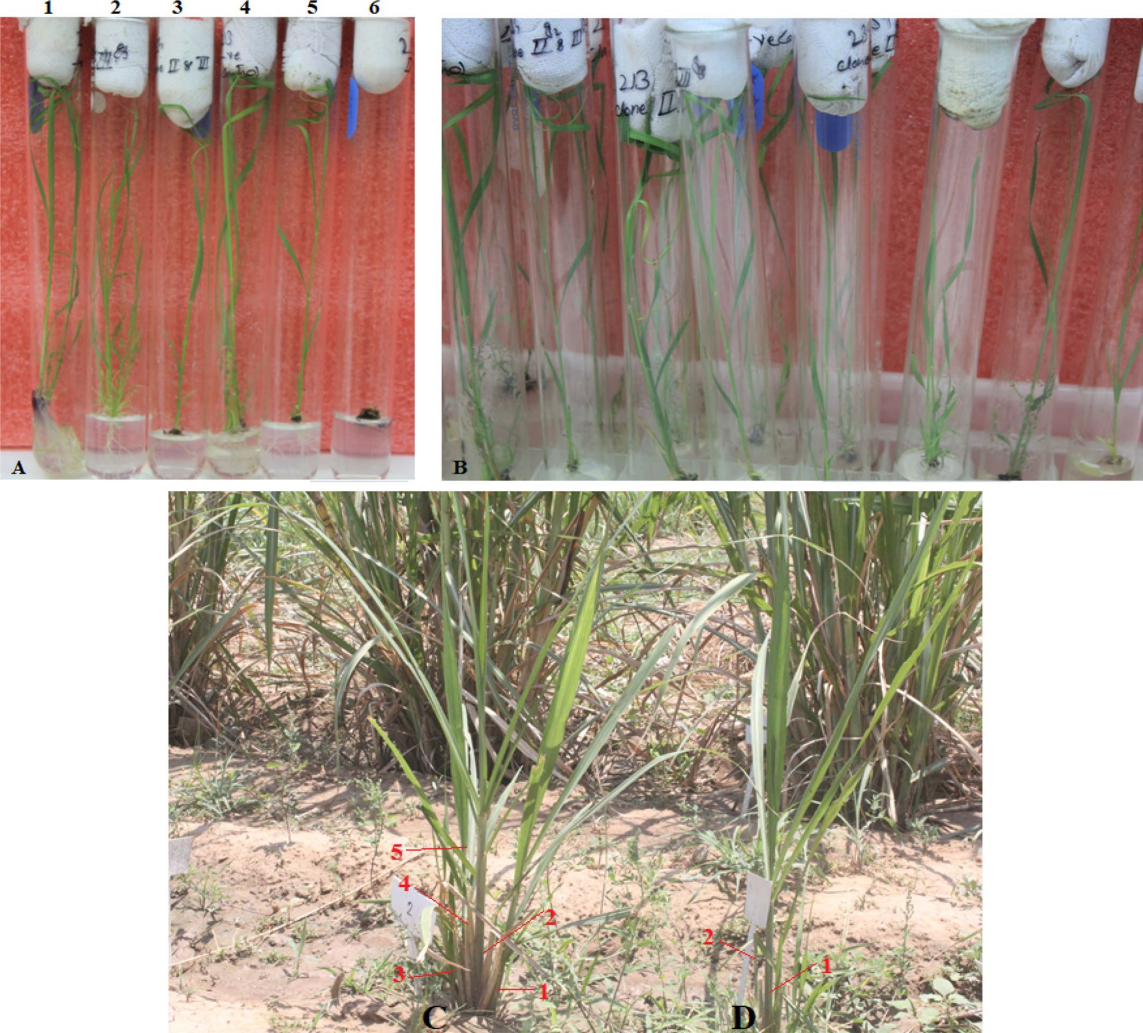
(**A**) Some selected transgenic lines were screened under hygromycin (100 mg/ml) selection media to evaluate putative transgenic lines accompanied with *ThSyGII (CEMB-SIG2)* on MS test tubes. Transgenic line 1–5 withstood hygromycin selection medium, survived and showed robust growth under selection medium. The line labelled with 6 is non-transgenic which could not tolerate hygromycin supplemented selection media (**B**). The Fig. 4B indicated control sugarcane plants without any selection media which showed maximum growth in the same conditions as provided to putative transgenic lines but without any inclusion of selection drug. The figure was cropped to enhance focus and to remove distractions in the figures. Different figures were taken in different numbers and in various groups but all shown plants in figures were captured at the same day to develop synchronizations in our data. The originally available plant figures were also provided in supplementary information file as figure S-4A & S-4B. (**C**). The juvenile sugarcane transgenic line (3 months old) positive for *ThSyGII* phenotypically shown better tillering and health. Red labels (1, 2, 3, 4, 5) represented the numbers of tillers developed in transgenic sugarcane lines. (**D**). The sugarcane plant shown in this diagram indicates the phenotypic behaviour of control sugarcane plant, Red labels (1, 2) in this picture are sufficed to indicate less number of tillers than transgenic sugarcane line. The cropped figure focused only two sugarcane lines including both transgenic and control while full field grown sugarcane was provided in supplementary information file as S-4C &S-4D.

Regenerated putative transgenic sugarcane lines were subjected to drug selection pertaining to antibiotic (hygromycin, 100 mg/ml) resistance in test tube MS zero media. The transgenic lines SIP32 and SIP33 could not survive in drug selection media showing the absence of *ThSyGII* construct in its genome. Plants SIP31, SIP34, SIP36, SIP37, SIP40, SIP41, SIP45, SIP46 and SIP48 survived indulging in selection media.The phenotypic data confirmed their resistance against hygromycin drug and could be seen in good health conditions. Figure 4A illustrated transgenic lines positive for*ThSyGII* gene construct. Similarly, Fig. 4B represented all transgenic plants grown under zero selection media and were shown inoptimum plant growth without the death of any single plant. Similarly, after 3 months, transgenic and control plants were shifted to field conditions as shown in Fig. 4C,D.

### Putative transgenic sugarcane lines show PCR amplifications and dot blotting indicating the integration of *ThSyGII (CEMB-SIG2)*

**Figure 5:**
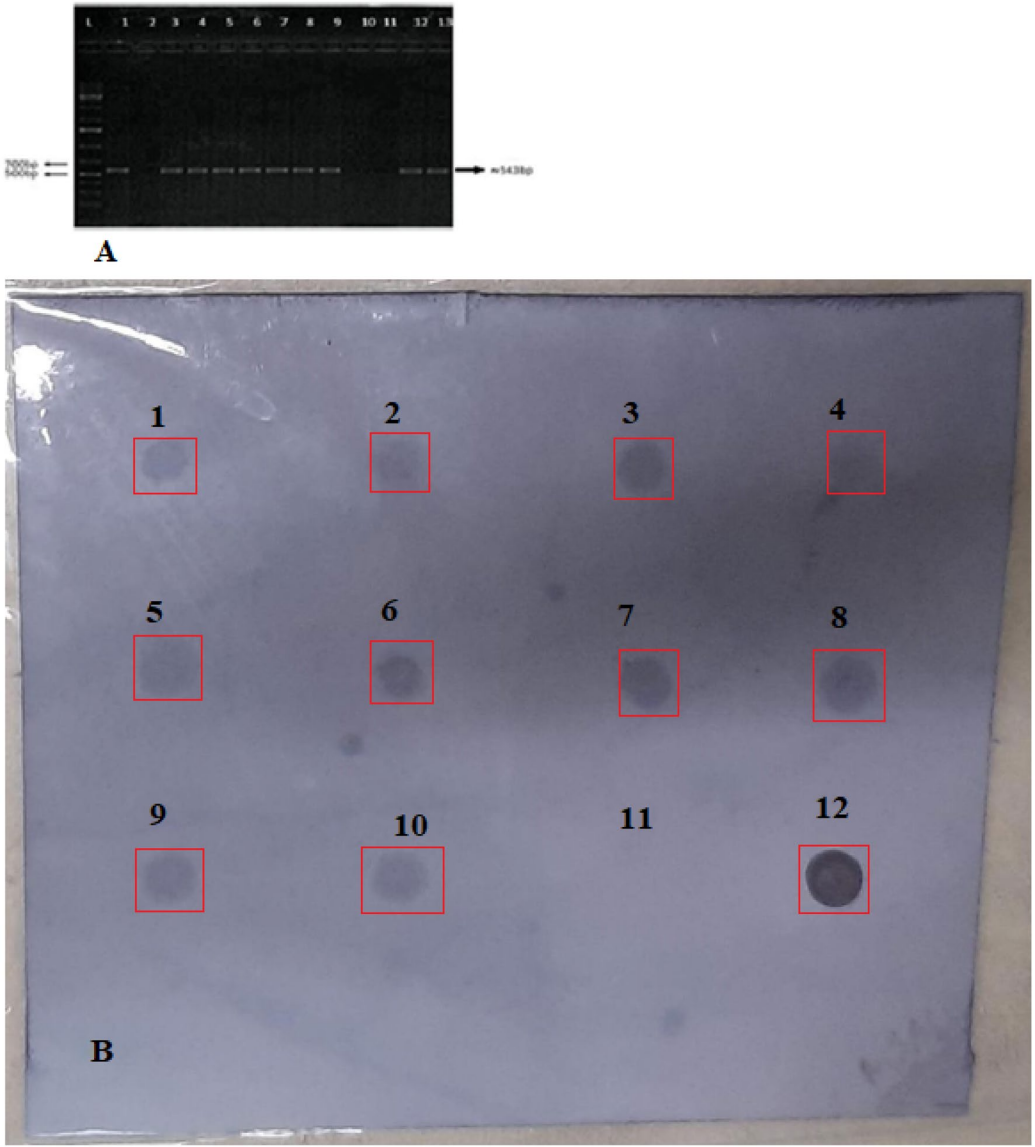
(**A**) PCR amplifications of positive plants indicating integration of *ThSyGII(CEMB-SIG2)* with 543 bp amplicon. Lane L ladder, 100 bp, lane 1 representing postive control, lane 2, negative control, lanes 3,4,5,6,7,8,9,12,13 show PCR positive lines while lanes 10 & 11 indicate PCR negative plants. The gel picture was cropped to exclude un-required raw gel bands not included in our study. The original uncropped gel pic was provided in supplementary information file as figure S-5A (**B**). Dotbot analysis inciating the blotting of all red boxes enclosed labelled selected PCR positive tarnsegnic lines are shown for all positive and negative control lines. The blotting spots 1–10 showed all transgenic sugarcane lines while 11 indicated negative control and 12 spot shows positive control. The membrane was covered with plastic to avoid further membrane blackening. Paint software tool was used for labelling and cropping targeted transgenic lines. The uncropped membrane edges shown RNA dot blot picture was also shown as supplementary part as figures S-5A & S-5B.

The figure 2C indicates the integration of PCR amplicon with 543 bp product size amplified byprimer specific sequences. The Fig. 5A,B showed in lanes 10 &11 negative sugarcane lines having no integration of transgene while clear bands at 543 bp showed the presence of *ThSyGII (CEMB-SIG2)*.

### Transgene *ThSyGII* exhibited triggered diversified pattern of expression levels in transgenic *Saccharum officinarum* lines

**Figure 6:**
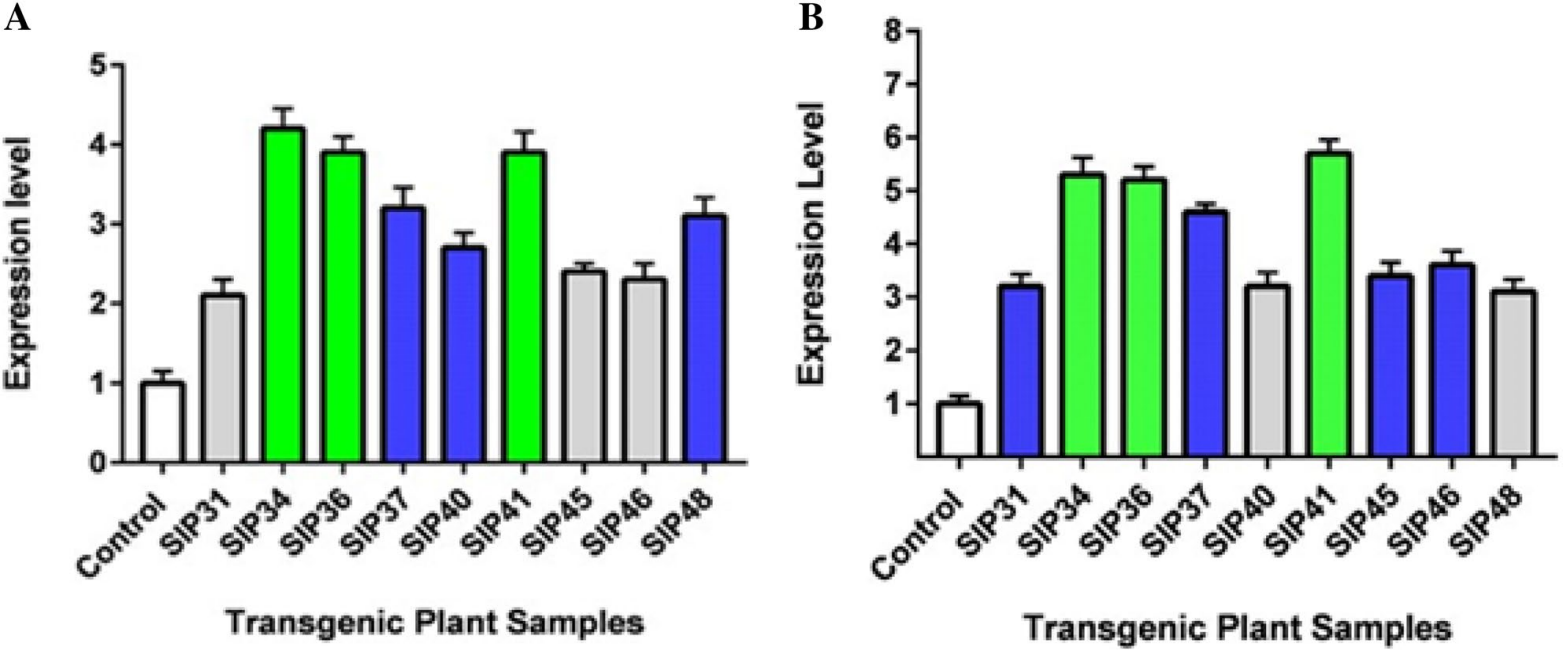
(**A**) *Trehalulose synthase geneII* (*ThSyGII*) expression levels of PCR positive transgenic lines extracted from leaf tissue samples in contrast to control lines (**B**) *ThSyGII* expression levels representing PCR positive transgenic lines in from stem tissues contrasting to control lines. Green color transgenic lines manifested highest expression, blue color transgenic lines conveyed medium level ThSyGII expression while grey lines indicated lowest gene expression levels. White colored sugarcane lines showed control non-transgenic lines.

Real Time PCR experiment of PCR positive sugarcane lines showed validation and produced expression profiles in transgenic lines. The transgene expression levels represented the activity levels of *ThSyGII* in different tissues ofplant. Our results showed that *ThSyGII* expressed tremendously in sugarcane leaves. The transgenic lines SIP34, SIP36 and SIP41 were graded at highest levels with 4.2, 3.9 and 3.8 fold expressions respectively than control lines. The *ThSyGII* transgenic lines SIP37, SIP48 and SIP40 exhibited 3.2, 3.0 and 2.8 times more expression respectively than control lines considered being at middle level. Moreover, remaining sugarcane lines SIP31, SIP45 and SIP46 could only indicate 2.1, 2.3 and 2.2 fold expression of *ThSyGII* respectively to control lines (Fig. 6A). The expression level of *ThSyGII* indicated expression profile obtained from stem tissues were extremely encouraging. The Fig. 6B indicated that transgene expression in stem tissues in transgenic lines SIP34, SIP36 and SIP41 boosted at 5.2, 5.1 and 5.8 fold high expression levels than control lines. Medium expression pattern originated from SIP31 (threefold), SIP 37 (4.8 fold) while SIP45 witnessed 3.1 times expression than control lines respectively. Transgenic lines, SIP46, SIP40, and SIP48 proclaimed 3.3, 3.1 and 3.2 fold expression levels than control lines (Fig. 6B).

### Stem mediated combinatorial promoters (PolyUbi + CmYMV) driven expression levels in transgenic sugarcane stalks

**Figure 7:**
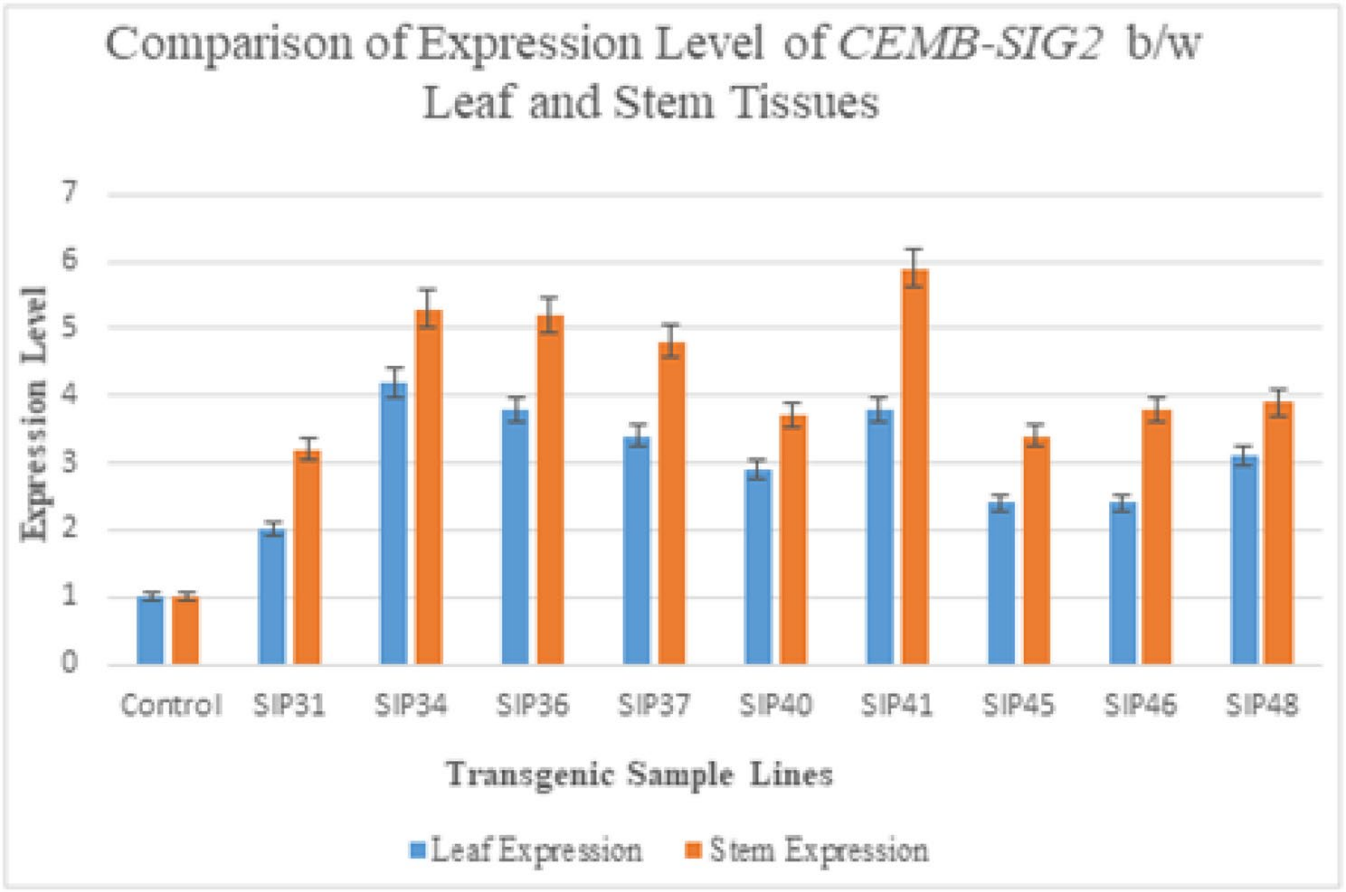
Comparative expression patterns of *ThSyGII* (*CEMB-SIGII*) gene in leaves and stalks. Blue bars exhibited *ThSyGII* expression levels in leaves while orange bars indicated *ThSyGII* expression levels in culm tissues (p < 0.05).

Transgenic sugarcane lines harboring *ThSyGII* intimated incompatible expression levels both in leaf and stem tissues. Our results indicate that all lines especially SIP36, SIP34, SIP37,SIP48 and SIP 46 showed increased *ThSyGII* expression levels. Highest difference level was observed in stem and leaf tissues in SIP46 stood at 77.2% more than leaf tissues. Percent expression level enhancement in transgenic lines occurring in culms was calculated separated and represented in Fig. 7. The percentage increase of *ThSyGII* expression level in SIP34, SIP36, SIP37, SIP40, SIP41, SIP45 and SIP48 was stood as 60%, 26.8%, 30.7%, 33.3%, 32.1%, 52.6%, 47.8% and 25.8% respectively in stalks rather than in leaf tissues. Lowest difference level of *ThSyGII* happened in SIP48 as shown in Fig. 7.

### Exploration of novel supersugar lines (SSL) with highest manifestation of SRP after 16 months of maturity

**Figure 8:**
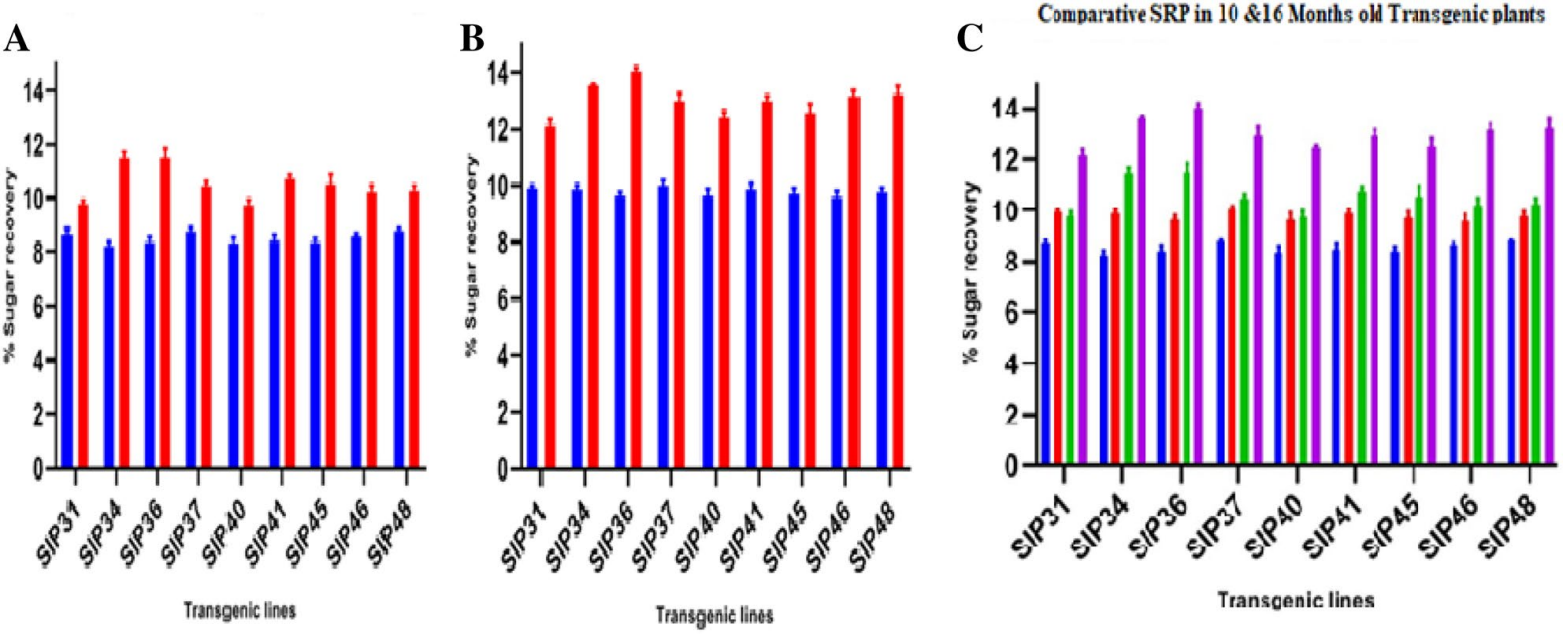
(**A**) The estimation of SRP after 10 months of maturity. (**B**) Estimated SRP after 16 months of plant maturity. Blue bars witnessed SRP levels in control lines while red bars indicate SRP levels in transgenic lines (**C**) Comparative SRP estimation after 10 and 16 months of maturity in transgenic and control lines. Blue bars represented 10 months old control lines, red bars indicate 16 months old control plants SRP estimations, green bars exhibited SRP levels in transgenic lines at 10 months stage whilepurple color bars are the representatives of 16 months old transgenic sugarcane lines.

The estimation of SRP by brix method showed a substantial increase in mature 16 months old sugarcane lines. Transgenic lines SIP46, SIP31 and SIP41 proclaimed 69.5%, 55% and 51.2% enhancement sugar contents respectively after 16 months than measurement at 10 months. Transgenic lines SIP37, SIP45 and SIP40 reflected 48.4%, 43.4% and 34.4% triggered SRP improvement respectively. Other lines SIP48, SIP36 and SIP34 indicated 25.8%, 30.7% and 29% increased SRP respectively after 16 months. Control non-transgenic lines only indicated 5% enhancement in SRP after 6 months. Figure 8 conveyed the comparative increase in SRP in different transgenic lines at different time intervals. Highest level of SRP boost was observed in six months in SIP46 (69.5%) while lowest SRP level 25.8% was recorded in SIP48 line.

### Segmentations of internodal novel TH production depicted by HPLC in SuperSugar line

**Figure 9:**
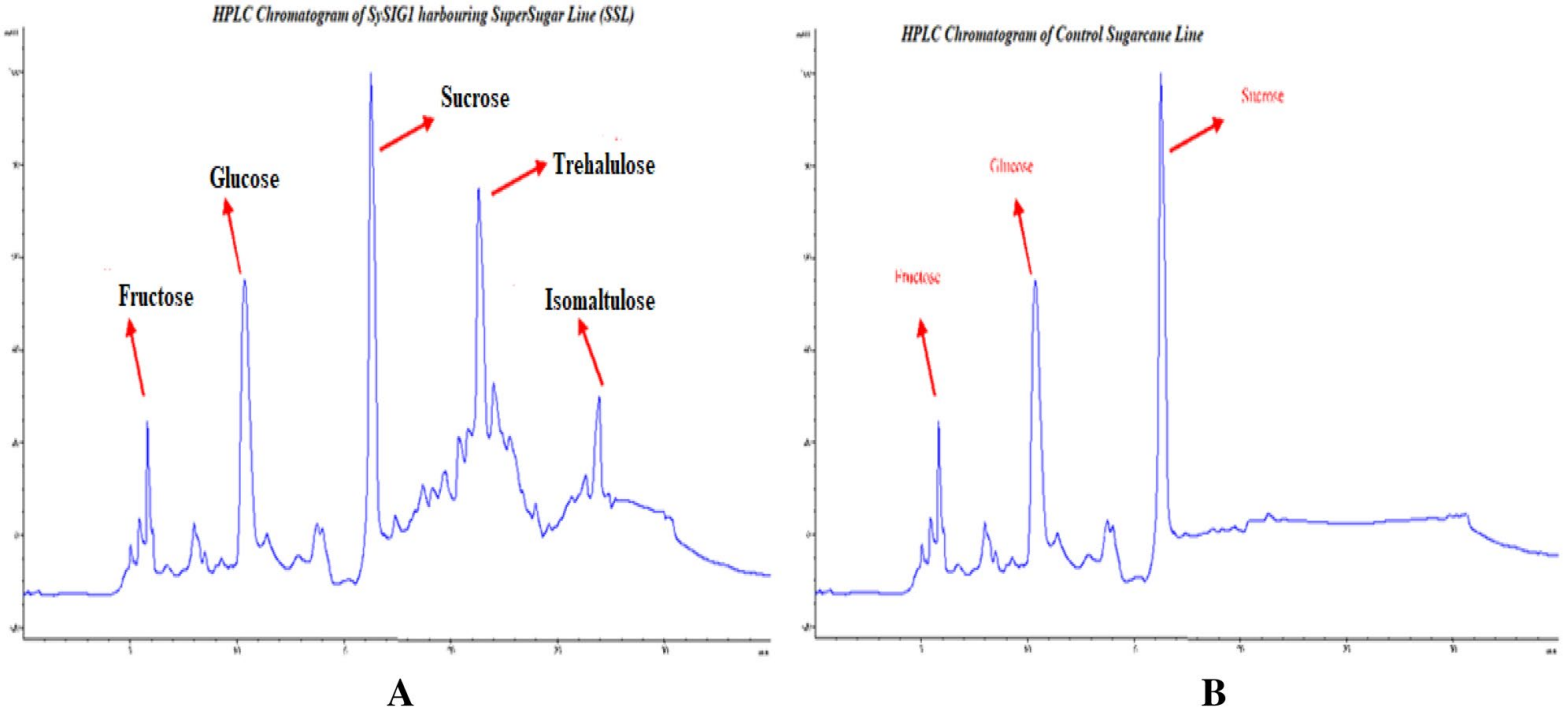
(**A**) The automatic HPLC based quantifications of various sugar contents obtained in different transgenic lines (**B**) HPLC based quantifications of control sugarcane lines employed in this study. Different peaks were shown by red arrows in both (**A**,**B**).

**Figure 10:**
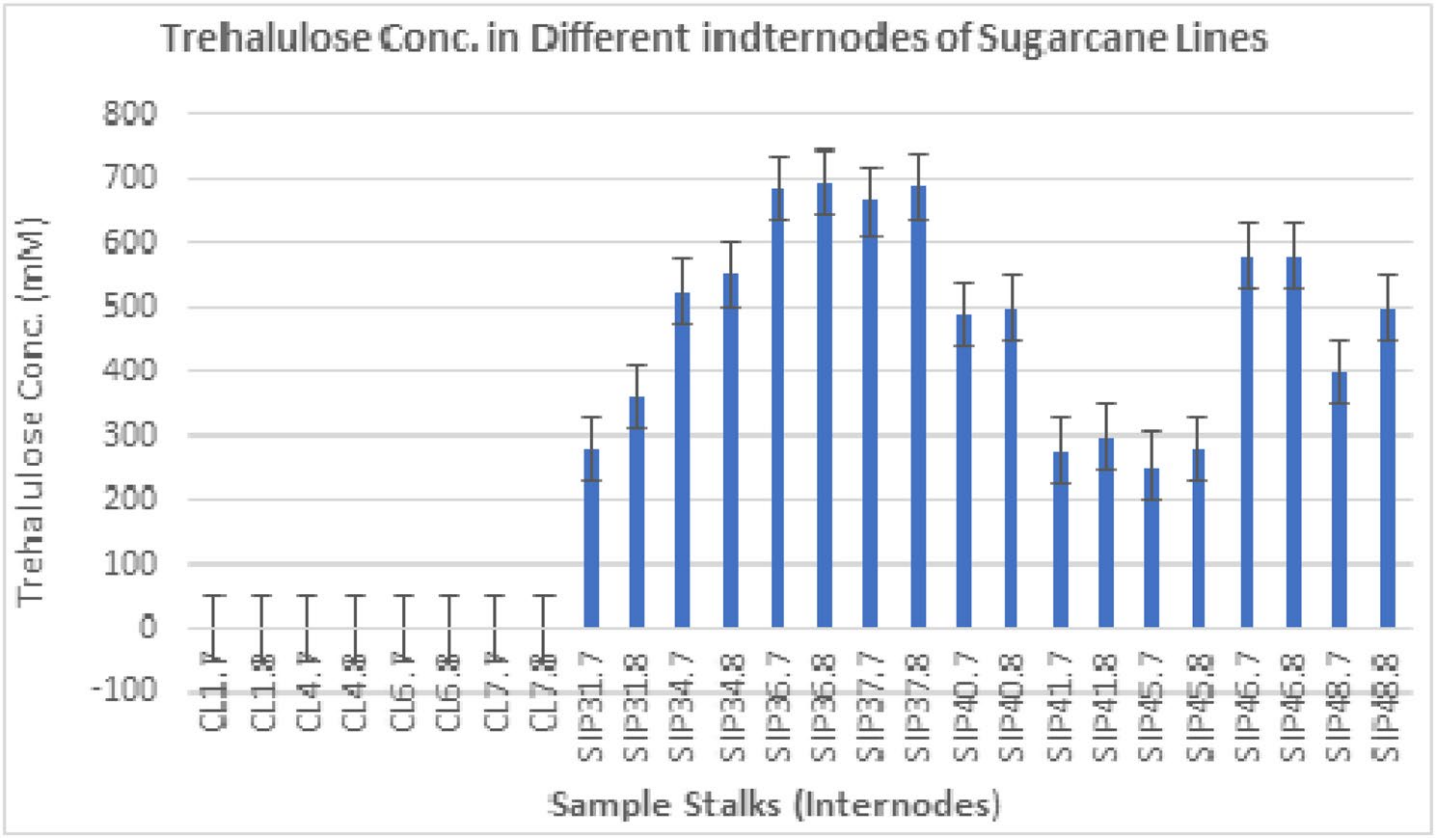
Graphical representations of TH contents quantified from HPLC based data from transgenic sugarcane lines contrasting to control lines.

HPLC was employed for ultimate estimation of TH concentrations in selected transgenic sugarcane lines. The quantification data obtained from HPLC experimentations as highest yielding TH producing transgenic sugarcane lines (Fig. 9A,B). Figure 10 communicated vivid phenotypic performance of various transgenic lines. Internodal samples from every single transgenic sugarcane line were quantified using HPLC instrumentation and TH output was calculated (Fig. 10). Transgenic sugarcane lines SIP36, SIP37 and SIP46 demonstrated maximum values crediting as Sweet booster lines (SBL) with 695 mM, 693 Mm & 690 mM TH contents in their mature internodes. Internodal stalk juice from SIP34, SIP40 and SIP48 resulted 356 mM, 498 mM and 497 m quantified TH components respectively. These lines stood at second grade category termed as bettersugar lines (BSL) while remaining SIP31, SIP41 and SIP45 exhibited 367 m,300 m and 287 m respectively nomenclature as Good sugar lines (GSL). Non-transgenic control sugarcane internodes (Fig. 10) nullified the presence of any TH contents due to the absence of *ThSyGII*.

### Estimation of different sugar contents in transgenic sugarcane line from HPLC quantifications

**Figure 11:**
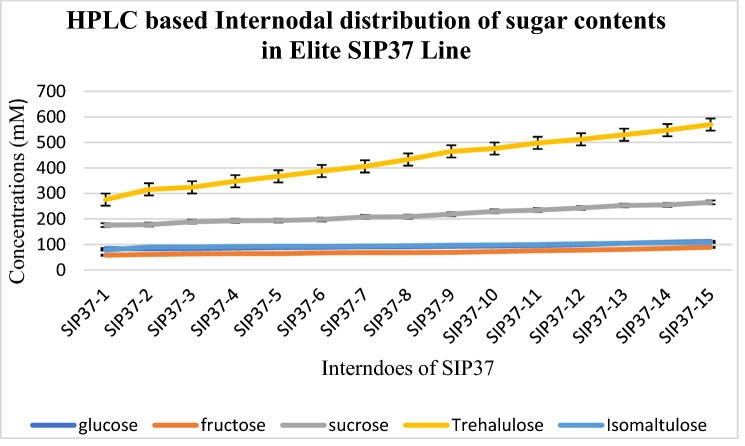
Graphical distribution of various sugarcane juice contents in different internodes of SIP37 sugar line. Yellow line (TH), grey line (SUC), blue line (IM), dark blue line (F) while orange line indicated FRU distribution across the whole sugarcane length.

Different sugar contents obtained in sugarcane juice included SUC, GLU, FRU, TH and IM were quantified by HPLC method as shown in various internodes of SIP37. The transgene *ThSyGII* harboring transgenic line SIP37 was one of the SBLsexhibited maximum SRP. Sugarcane juice estimation witnessed that highest quantity of TH was produced and harvested in mature internodes. The level of TH increased directly from immature to mature interndoes. Immature interndoes exhibited lowest TH in interndoe SIP31-1 which was 276 mM while highest TH was obtained from SIP37-15. The SUC level in SIP37-1 interndoe was 176 mM and it increased to 265 mM in matured SIP37-15. The least amount of FRU was found in SIP37-1, 58 mM boosted to maximum 89 mM in SIP37-15. Similarly the distributions of IM and GLU were also quantified by HPLC based method as depicted in Fig. 11.

## Discussion

The examination modified *ThSyGII* expression in *Saccharum officinarum* L. and its contribution towards enhancement of SRP was the aim of this proposed study. The hypothesis was based on the assumption that modifications and integration of *ThSyGII* in sugarcane might produce different expression profiles under combined promoter system, leading to encroachments of healthful sugar contents in sugarcane. Three out of nine transgenic lines, SIP36, SIP37 and SIP46 demonstrated accumulation of SUC isomer quantity > 600 mM after 16 months of maturity. All nine tested sugarcane lines proved promising and credible hallmarked with newly embedded TH. As obvious, control plants did not exhibit any trace of TH due to the absence of *ThSyGII*. Although, post transcriptional gene silencing (PTGS) is very common issue in sugarcanedue to complexity of its genome poses serious challenges for transgene stability^31^. Different SIGs contain antagonistic mechanism resisting PTGS. Hence transgene stability is not compromised and remains intact as reported in previous studies^32–34^. Gene silencing was the potential threat in present work as it exploited pUbi promoter, a partial target of gene silencing^35^ but not a single observation related to silencing event, transcriptional or post transcriptional gene silencing was seen. Not only pUbi exhibited resistance against PTGS but combinatorial modified promoter system in current research work also proved successful^36^. This unique characteristic of *ThSyGII* triggered enhancements of whole sugar contents in transgenic sugarcane crop^37^.Hence the hypothesis use of two combined promoters may enhance transgene expression level, seems significant in our study. Three high TH yielding SBLs are strong candidate as SUC alternatives in food industries^26^. High yielded TH concentrations down to stalks increased SRP in transgenic sugarcane lines as already reported in previous reports^38–40^ but another report negated that fact and described antagonisticnotion^41^. Our research work validatedthat SRP from sugarcane could be augmented significantly by employing SIGs with efficient cloning strategy. All transgenic lines not only produced enhanced quantity of TH but also consolidated final SRP partitioned in different internodal regions. Previously pUbi was considered relatively a weak promoter against PTGS effects but current workidentified that PTGS could be neutralizedafter employing combinatorial dual promoters integrated with VTS^42^. Another recent study also investigated the role of different promoters, degree of their expression in genotypes KQ228 and Q208, obtained fromleaf and internodal tissues. Expression level of SUC phosphate synthase (SPP) in gene family was higher in leaves than in mature internodal samples. Meanwhile in another similar gene family degree of expression by SPP isoforms1 &2 were also evaluated. That study revealed sometimes expression get increased in roots as compared to leaves and stalks^43^. In our research work, pUbi and CmYMV promoters combined to increase *ThSyGII* expression in stalks as compared to leaves. Although expression in leaves was also good but their combinations become highly advantageous leading to enhanced SRP. Current research work did not focus on estimation of gene expression in the roots as reported in previous studies^44^. The primary objective was to harvest boosted SRP in sugarcane juice obtained from stalks tissues. Increased degree of TH enhancement down to lower internodes from top to base was observed in our work.This fact was the result of continual availability of *ThSyGII* driven under combined constitutive modified promoters across the sugarcane plant. An efficient catalytic performance indicating immediate remarkable TH assembly was observed before its deliverance to belligerent vacuole. Hence plants are unable to metabolize SUC isomers^24^. Achieving triggered yield and purified TH containing valued compound required optimizing mechanism of developmental gene expression, stability related to TH synthase in localized vacuoles so that increased TH contents might be progressive to harvested sugarcane stalks.

This study also materialized high *ThSyGII* expression level in sugarcane stalks than leaf tissues. All transgenic events exhibited substantial degree of augmentations inexpression levels in sugarcane stalks contrasting to leaf tissues. The present research initiative also validated expression results obtained in another research work^26^. Transformants inducing *ThSyGII* produced IM comprising 18% of the overall SUC isomer in sugarcane stem at all possible locations, this proportion indicated stability in homogeneity which existed between IM and TH in sugarcane internodes. The SRP data highlighted the performance of selected sugarcane lines yielding significant increase in whole sugar contents in *Saccharum officinarum* L. Transgenic lines SIP34, SIP36, SIP37 and SIP46 produced more than 14% SRP in contrast to 9% in control lines. These SBLs showed 55.5% more sugar contents than control lines. All selected sugarcane lines were subjected to internodal estimation of SRP,screened out internode 12 from all sugarcane lines supersedingall upper internodes withmaximum TH contents (> 700 mM) ultimately materialized to super SRP (> 900 mM) (Fig. 9). Internodes 11 and 12 of all selected transgenic lines yielded maximum SRPin comparison to lower SRP happened in juvenile internodes. Similar reports indicated that better SRP can be obtained in internode number 10 as appeared in current studies^45^. Another interesting feature was also analyzed and experienced in all nine lines exhibiting TH > 700 mM. It not only harvested TH but also produced 14% IM as SUC isomer. There was not any change in SUC in addition to productions of TH and IM. The proportion of TH and IM in SBLsconsisted of 77% of total SRP levels of SUC remains intact. The concentrationsof SUCin both control and transgenic sugarcane lines revealedidentical quantifications, meaning in addition to isomerization of SUC to TH and IM, it did not show any negative effect in overall cellular SUCconcentrations. Previous studies involved with SUC isomerization witnessed the production of only 40% TH from total sugar contents 75% lesser than found in our study. Such huge difference appeared due to the exploitation of combination of leaf and stem tissue specific promoters^46^. Such novel phenomenon was believed to occur due to role of vacuole targeted signal peptides derived from sweet potato, directing all possible protein manufacturing and storage into stem vacuolar region. As vacuole has hostile environment against SI, SUC remains safe from SI function and adds additional TH in total SRP. Cytoplasmic SUC isomerized into TH (major) and IM (minor) by the action of combined modified promoters driven *ThSyGII* gene^47,47^. Preliminary growth of sugarcane lines in small pots under small greenhouse conditions was at lower degrees as compared to first vegetative growth from setts in the open field conditions. Experienced sugarcane breeders affirmed that close lines prefer obtaining biomass than adding SRP. It was evident that sugarcane lines grown under open field conditions, sharing similar conditions showed no significant variability in SRP^48^**.** Epigenetic modifications and variability in tissue culture conditions also played pivotal role in SRP which need scientific attention^49,50^. A broad spectrum of relationship between TH and SRP in sugarcane was also learnt from this study. Significant increasein TH and IM productions (> 700 mM) caused remarkable 77% augmentations in total SRP. Poor SRP producing GSLs (SIP31, SIP41) yielded 24% high SRP than control plants at 16 months of age. Moreover transgenic lines exhibited robust vigor, better vegetative growth, increased level of photosynthetic efficiency and strong phenotypic morphological features. Increased field plot experiments need to be performed to reach real estimation of biomass gain^41^ so that gene stability may be determined in further vegetative generations.

## Supplementary Information

Supplementary Information.

## Data Availability

The datasets generated and/or analyzed during the current study are available in the manuscript.

## References

[1] Awan MF (2019). Evaluation of genotypic and hormone mediated callus induction and regeneration in sugarcane (*Saccharum officinarum* L.). Int. J. Bot. Stud..

[2] Iqbal, M. *et al.* Genetic variability of sugarcane genotypes for red rot. *Genet. Mol. Res.19*, gmr16039978. (2018).

[3] Rae AL, Perroux JM, Grof CP (2005). Sucrose partitioning between vascular bundles and storage parenchyma in the sugarcane stem: a potential role for the ShSUT1 sucrose transporter. Planta.

[4] Anwar Z, Gulfraz M, Irshad M (2014). Agro-industrial lignocellulosic biomass a key to unlock the future bio-energy: A brief review. J. Radiat. Res. Appl. Sci..

[5] Misra, V. *et al*. Sugar transporters, sugar-metabolizing enzymes, and their interaction with phytohormones in sugarcane. *J. Plant Growth Regul.* 1–14 (2022).

[6] Clemens RA (2016). Functionality of sugars in foods and health. Compr. Rev. Food Sci. Food Saf..

[7] Sawale PD (2017). Isomaltulose (palatinose)—An emerging carbohydrate. Food Biosci..

[8] Song, C. W. *et al.* Microbial production of 2, 3-butanediol for industrial applications.*J. Ind. Microbiol. Biotechnol.* **46**, 1583–1601 (2019). 10.1007/s10295-019-02231-0 31468234

[9] Soliman, H. I. Molecular cloning of sucrose isomerase gene and agrobacterium-mediated genetic transformation of potato (*Solanum tuberosum* L.) plants. *Int. J. Environ. Agric. Biotech.* **3**, 264352 (2018).

[10] Ghosh A (2016). Progress toward isolation of strains and genetically engineered strains of microalgae for production of biofuel and other value added chemicals: a review. Energy. Convers. Manag..

[11] Centeno-Leija S (2022). Mining for novel cyclomaltodextrin glucanotransferases unravels the carbohydrate metabolism pathway via cyclodextrins in Thermoanaerobacterales. Sci. Rep..

[12] Gangoiti J (2020). Synthesis of novel α-glucans with potential health benefits through controlled glucose release in the human gastrointestinal tract. Crit. Rev. Food Sci. Nutr..

[13] W. Patrick, J., C. Botha, F. & G. Birch, R. Metabolic engineering of sugars and simple sugar derivatives in plants. *Plant Biotechnol. J.* **11**, 142–156 (2013). 10.1111/pbi.12002 23043616

[14] Allan MC, Rajwa B, Mauer LJ (2018). Effects of sugars and sugar alcohols on the gelatinization temperature of wheat starch. Food Hydrocoll..

[15] Vuong TV, Master ER (2022). Enzymatic upgrading of heteroxylans for added-value chemicals and polymers. Curr. Opin. Biotechnol..

[16] Agarwal N, Narnoliya LK, Singh SP (2019). Characterization of a novel amylosucrase gene from the metagenome of a thermal aquatic habitat, and its use in turanose production from sucrose biomass. Enzyme Microb. Technol..

[17] Yin X (2022). A novel solvothermal biorefinery for production of lignocellulosic xylooligosaccharides, fermentable sugars and lignin nano-particles in biphasic system. Carbohydr. Polym..

[18] Wu L, Birch RG (2005). Characterization of the highly efficient sucrose isomerase from Pantoea dispersa UQ68J and cloning of the sucrose isomerase gene. Appl. Environ. Microbiol..

[19] Liu L, Yu S, Zhao WA (2021). Novel sucrose isomerase producing isomaltulose from raoultella terrigena. Appl. Sci..

[20] Liu L (2021). Studies on biological production of isomaltulose using sucrose isomerase: Current status and future perspectives. Catal. Letters..

[21] Zhang P (2018). High and efficient isomaltulose production using an engineered Yarrowia lipolytica strain. Bioresour. Technol..

[22] Docimo T (2020). Physiological, biochemical, and metabolic responses to short and prolonged saline stress in two cultivated cardoon genotypes. Plants..

[23] Luo Z (2020). Enhancing isoprenoid synthesis in Yarrowia lipolytica by expressing the isopentenol utilization pathway and modulating intracellular hydrophobicity. Metab. Eng..

[24] Liu G (2021). Stem vacuole-targetted sucrose isomerase enhances sugar content in sorghum. Biotechnol. Biofuels.

[25] Zawawi N (2022). Unique physicochemical properties and rare reducing sugar trehalulose mandate new international regulation for stingless bee honey. Food Chem..

[26] Damaj MB (2020). Unprecedented enhancement of recombinant protein production in sugarcane culms using a combinatorial promoter stacking system. Sci. Rep..

[27] Maraphum K (2020). Spatial mapping of Brix and moisture content in sugarcane stalk using hyperspectral imaging. J. Near Infrared. Spectrosc..

[28] Freitas, P. *et al*. Determination of phenolic acids in sugarcane vinasse by HPLC with pulse amperometry. *J. Anal. Methods. Chem.* **2018** (2018). 10.1155/2018/4869487 PMC5828258 29600112

[29] Daniel J (2017). Fibre digestibility and its relationships with chemical and morphological traits in thirty-two sugarcane varieties. Grass Forage Sci..

[30] Audilakshmi S (2010). Inheritance of sugar concentration in stalk (brix), sucrose content, stalk and juice yield in sorghum. Biomass Bioenergy..

[31] Peres, A. L. *et al*. Gene silencing using artificial miRNA in sugarcane. *Trop. Plant Biol.* 1–10 (2022).

[32] Otto R (2022). Sugarcane pre-sprouted seedlings: A novel method for sugarcane establishment. Field Crops Res..

[33] Kamwilaisak, K. *et al.* Estimation of sugar content in sugarcane (Saccharum spp.) Variety Lumpang 92–11 (LK 92–11) and Khon Kaen 3 (KK 3) by Near Infrared Spectroscopy. *Eng. J.* **25**, 69–83 (2021).

[34] Anur RM (2020). Overexpression of sucrose phosphate synthase enhanced sucrose content and biomass production in transgenic sugarcane. Plants..

[35] Widyaningrum S (2021). Induction of resistance to sugarcane mosaic virus by RNA interference targeting coat protein gene silencing in transgenic sugarcane. Mol. Biol. Rep..

[36] Ali S, Kim WC (2019). A fruitful decade using synthetic promoters in the improvement of transgenic plants. Front. Plant Sci..

[37] Li Y (2022). Expression profiling and MicroRNA regulatory networks of homeobox family genes in sugarcane *Saccharum spontaneum* L. Int. J. Mol. Sci..

[38] Gabriel C (2021). Genetic manipulation of trehalose-6-phosphate synthase results in changes in the soluble sugar profile in transgenic sugarcane stems. Plant Direct..

[39] de Oliveira L (2022). Bioinformatic analyses to uncover genes involved in trehalose metabolism in the polyploid sugarcane. Sci. Rep..

[40] Hu X (2020). Genome-wide analysis of the trehalose-6-phosphate synthase (TPS) gene family and expression profiling of ScTPS genes in sugarcane. Agron..

[41] Aguiar A (2021). Sugarcane straw as a potential second generation feedstock for biorefinery and white biotechnology applications. Biomass Bioenergy.

[42] Khan N, Bano AM, Babar A (2020). Impacts of plant growth promoters and plant growth regulators on rainfed agriculture. PLoS ONE.

[43] Ma, X. *et al*. An organ‐specific transcriptomic atlas of the medicinal plant Bletilla striata: Protein‐coding genes, microRNAs, and regulatory networks. *The Plant Genome*. e20210 (2022). 10.1002/tpg2.20210 PMC12807252 35475547

[44] Ghosh, S. & Dey, G. Biotic and abiotic stress tolerance through CRISPR-Cas mediated genome editing. *J. Plant Biochem. Biotechnol*. 1–12 (2022).

[45] Verma, K. K. *et al*. Impact of agroclimatic variables on proteogenomics in sugar cane (*Saccharum* spp.) plant productivity. *ACS Omega*. **7**, 22997–23008 (2022). 10.1021/acsomega.2c01395 PMC9280927 35847309

[46] Wang J (2021). Identification and analysis of stem-specific promoters from sugarcane and energy cane for oil accumulation in their stems. Glob. Change Biol. Bioenergy.

[47] Banerjee N (2022). Identification of microRNAs involved in sucrose accumulation in sugarcane (*Saccharum* species hybrid). Plant Gene.

[48] Singh R (2021). Hydrothermal pretreatment for valorization of genetically engineered bioenergy crop for lipid and cellulosic sugar recovery. Bioresour. Technol..

[49] Ghosh A, Igamberdiev AU, Debnath SC (2021). Tissue culture-induced DNA methylation in crop plants: A review. Mol. Biol. Rep..

[50] Silva P (2018). Fingerprint targeted compounds in authenticity of sugarcane honey—An approach based on chromatographic and statistical data. Lwt..

