# Supplementary material for: Enhancement of healthful novel sugar contents in genetically engineered sugarcane juice integrated with molecularly characterized ThSyGII (CEMB-SIG2)
Source: Sci Rep. 2022 Nov 3;12:18621. doi: 10.1038/s41598-022-23130-y (PMC9633787; doi:10.1038/s41598-022-23130-y)

**Supplementary Information File**

**Figure S-2A**

**
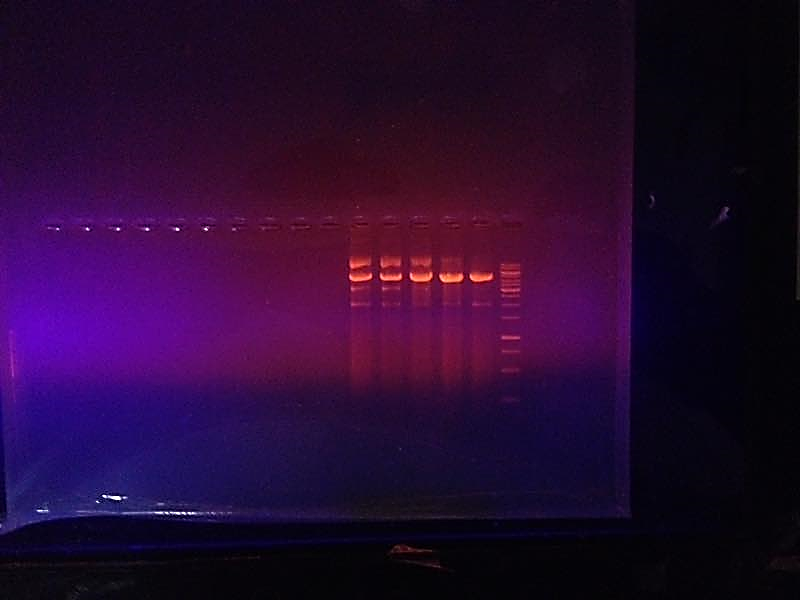
**

**Figure S-2B**


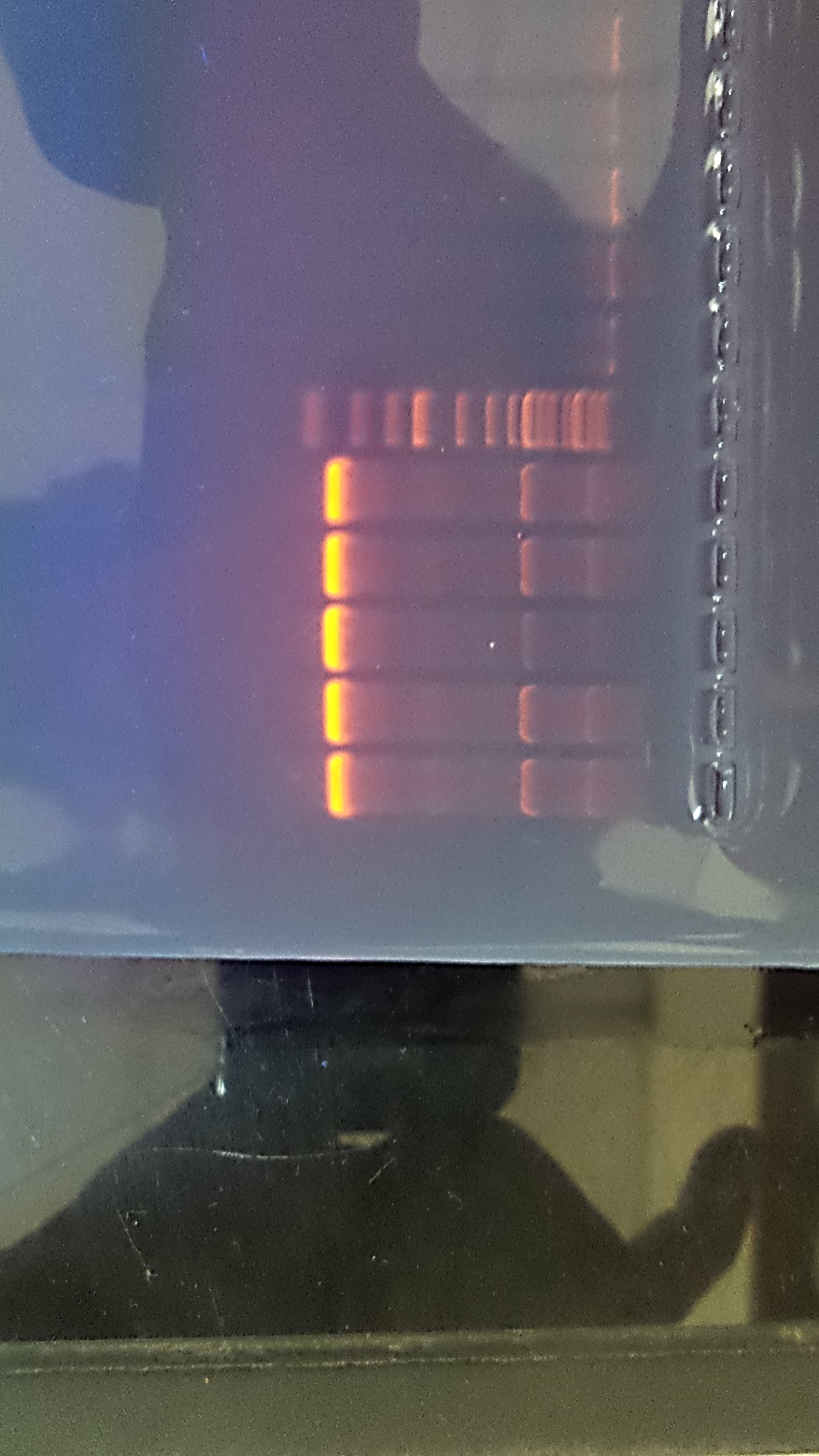


**Figure S-2C**

**
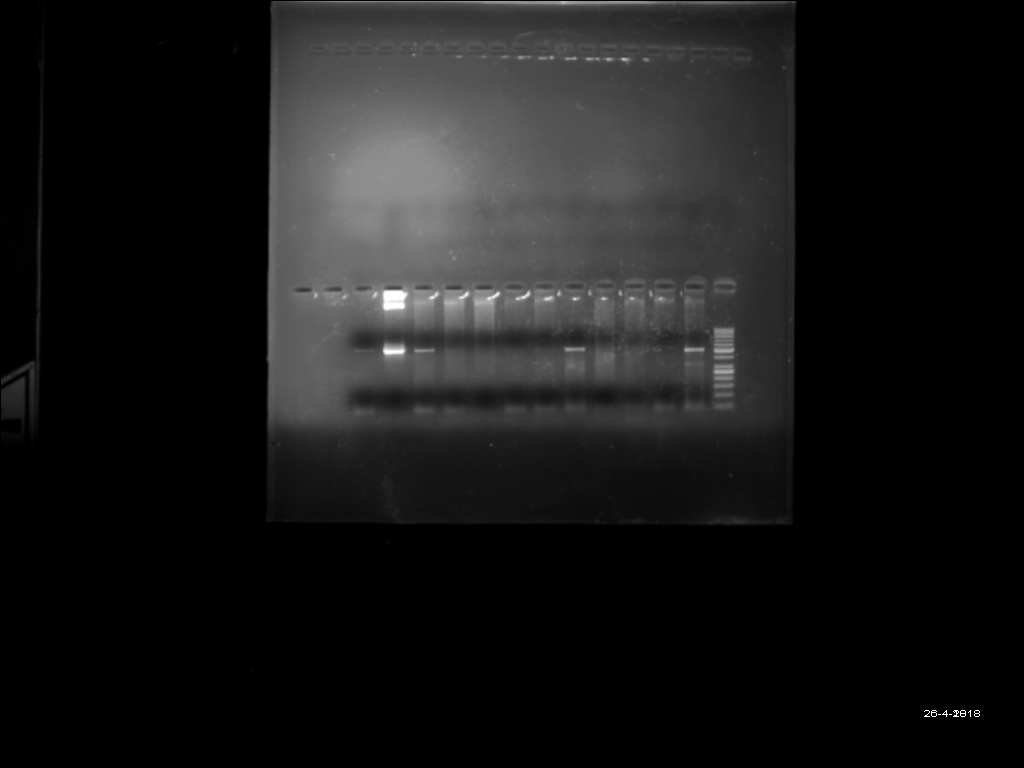
**

**Figure S-3A**


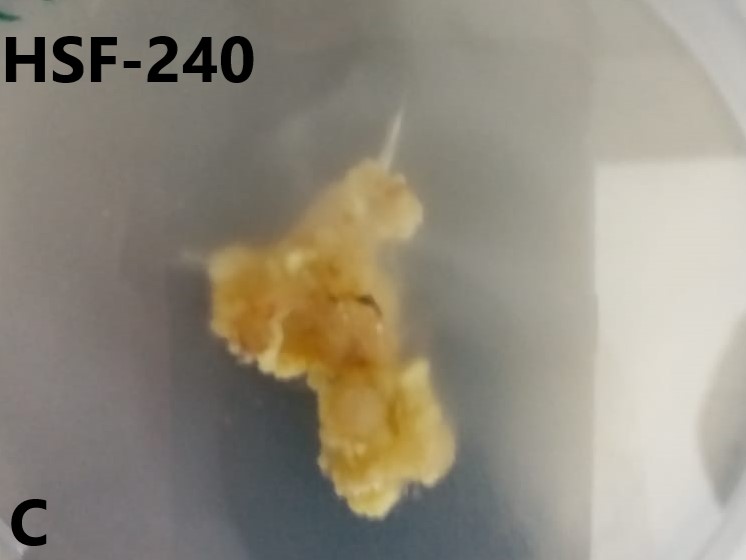


**Figure S-3B**


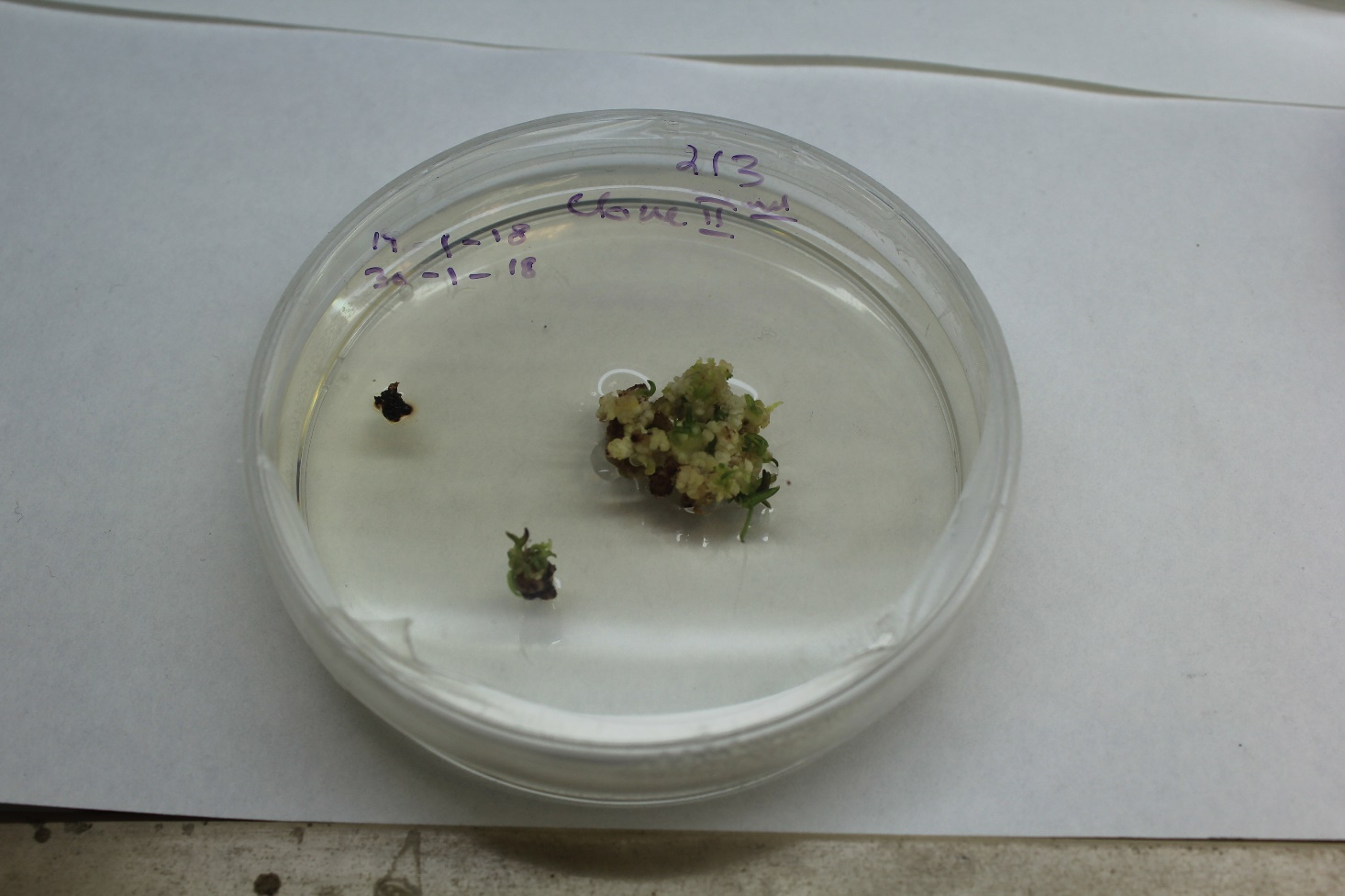


**Figure S-3C**


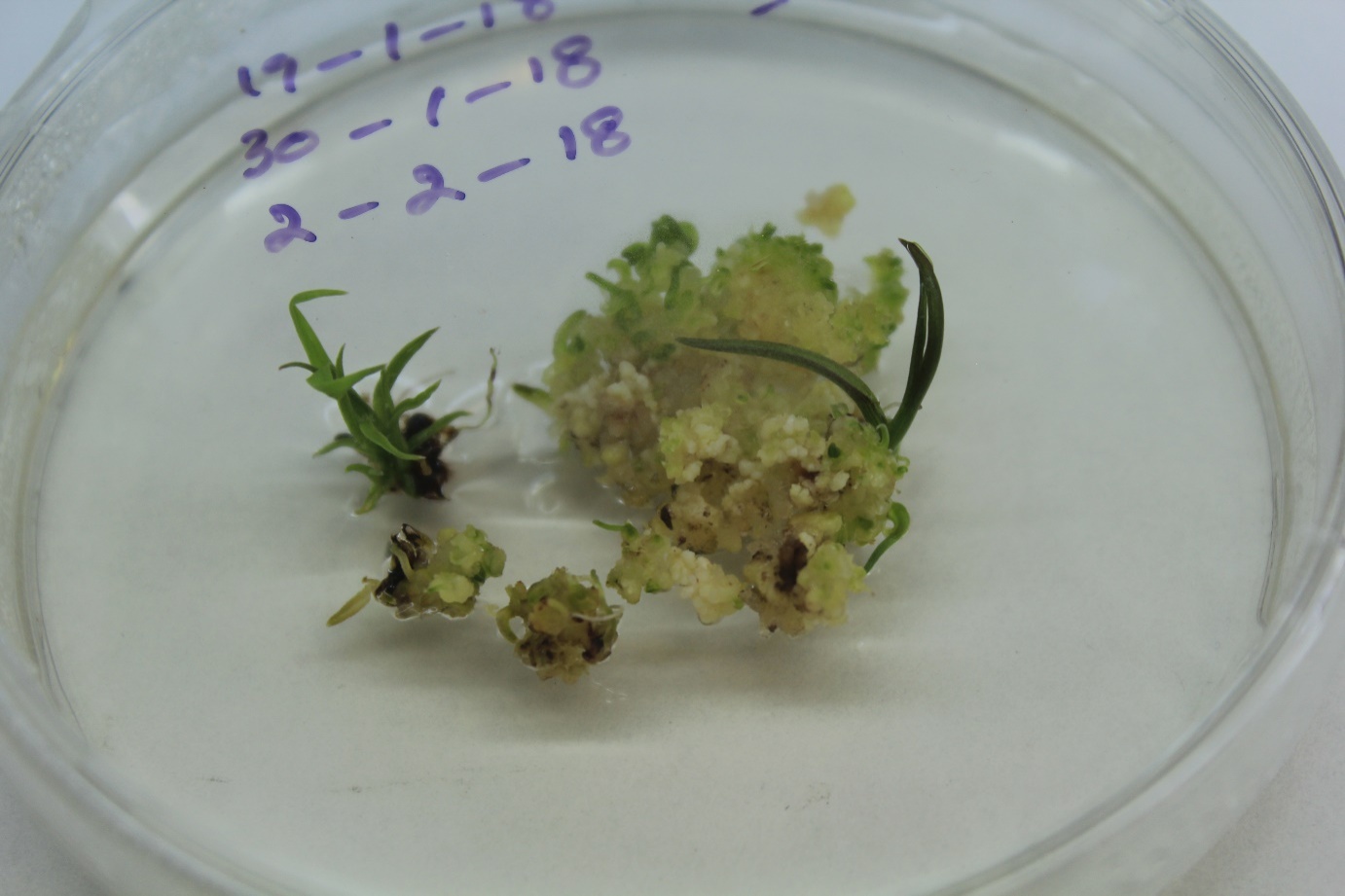


**Figure S-3D**


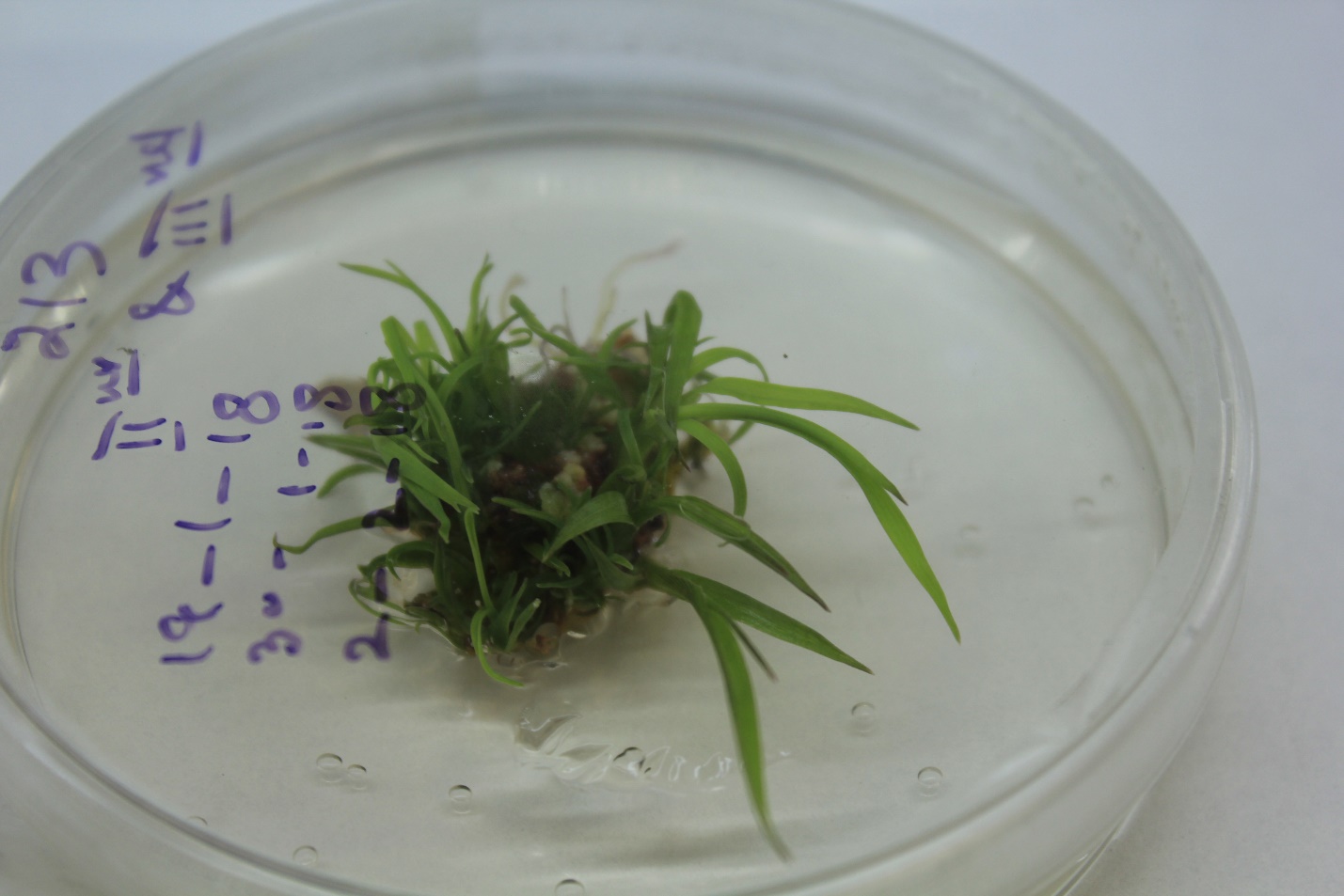


**Figure S-4A**


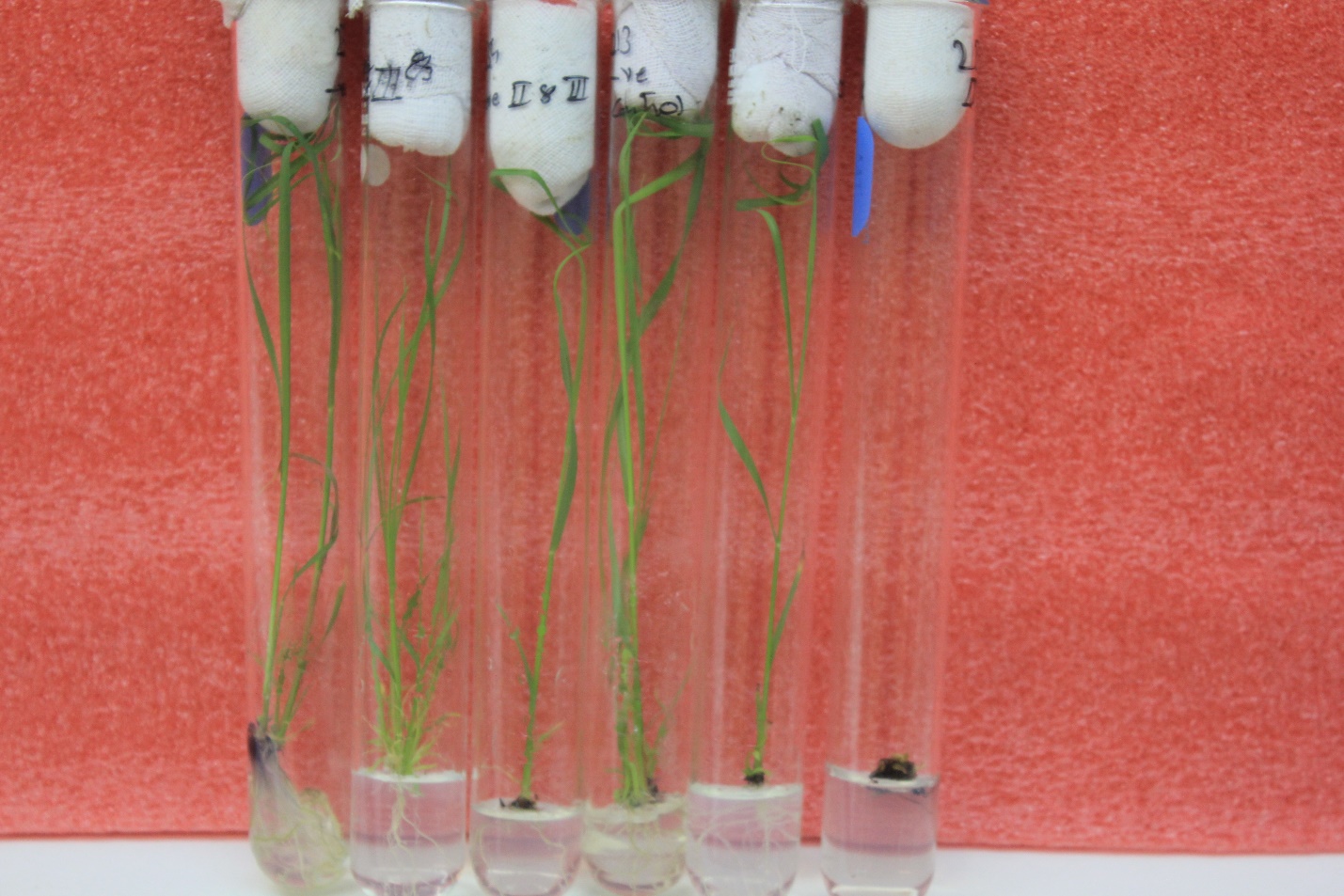


**Figure S-4B**


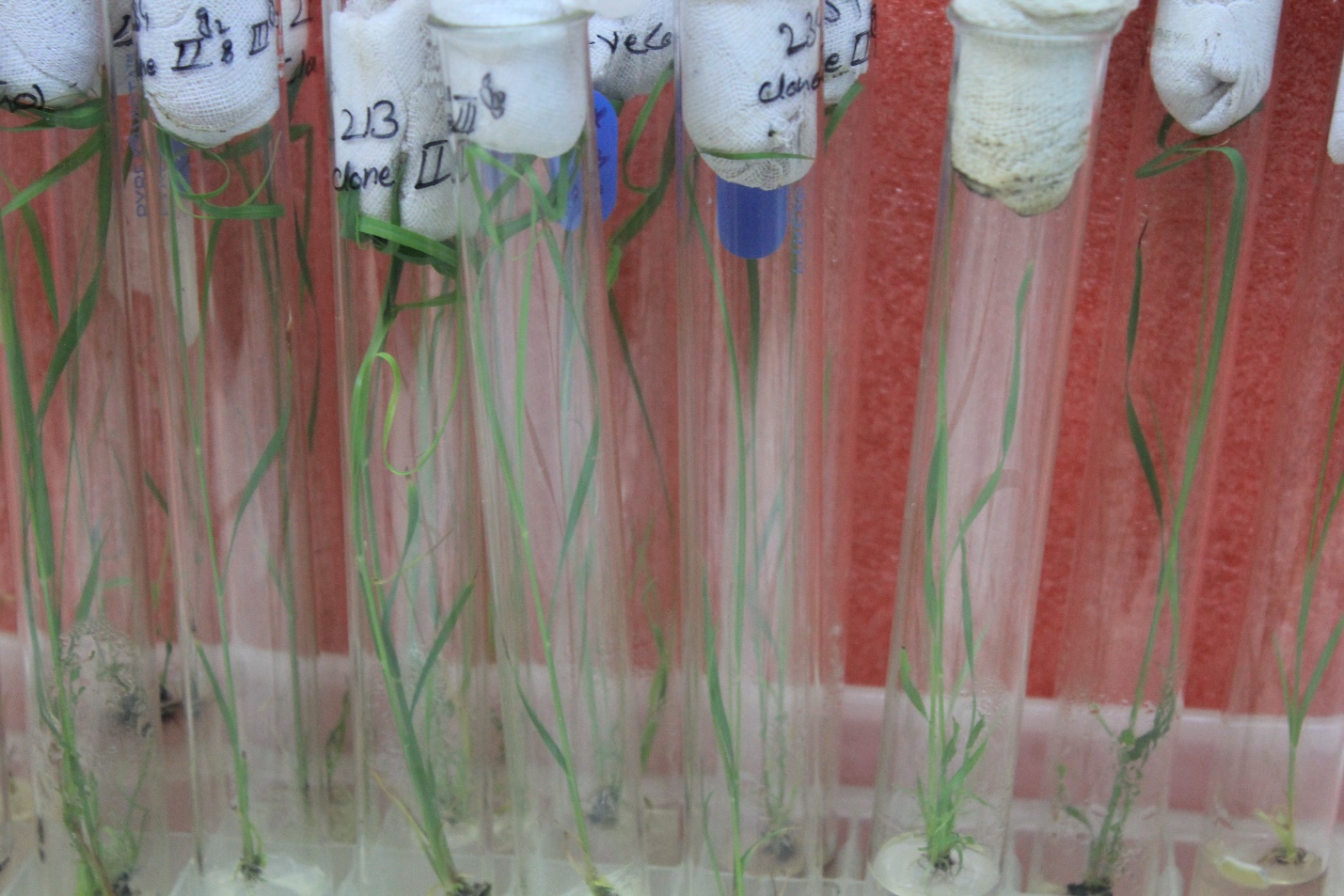


**Figure S-4C & 4D**


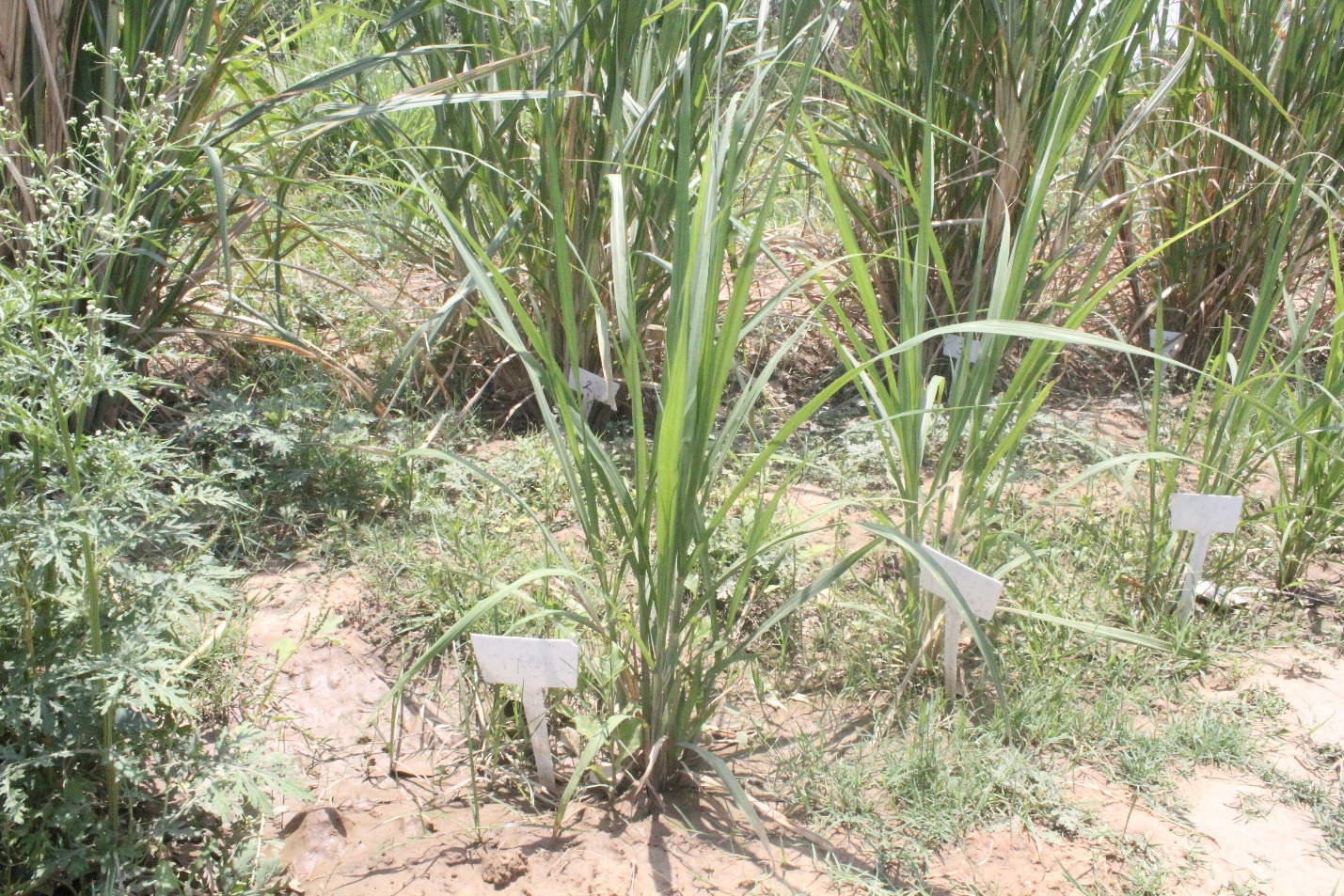


**Figure S-5A**


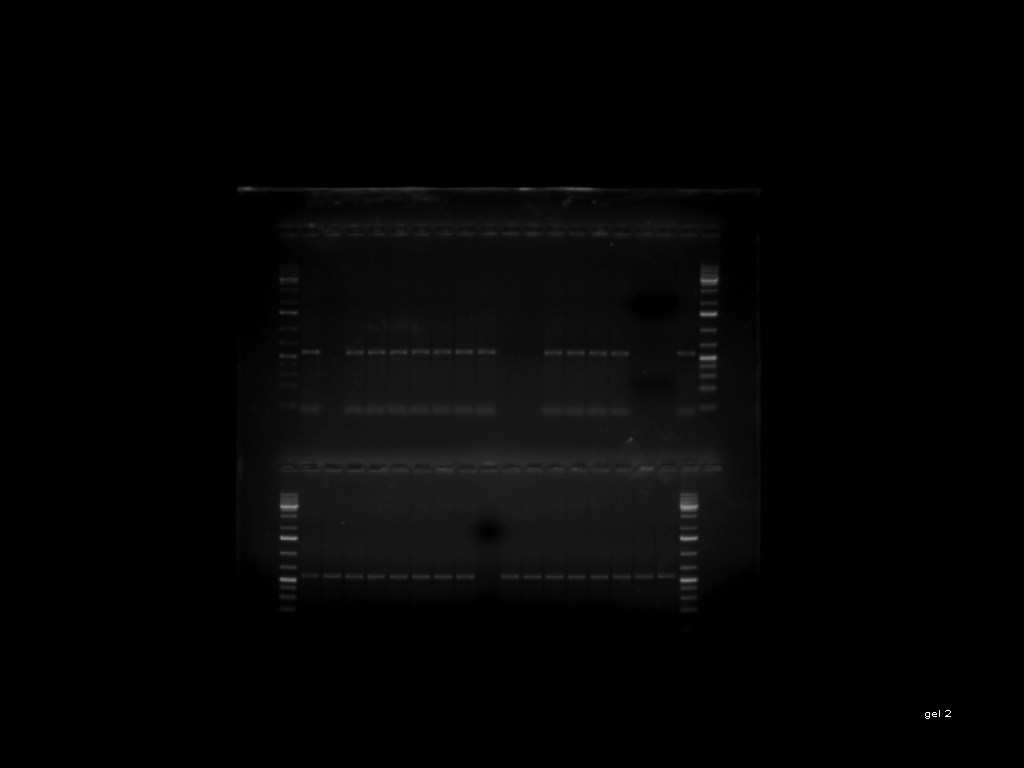


**Figure S-5B**


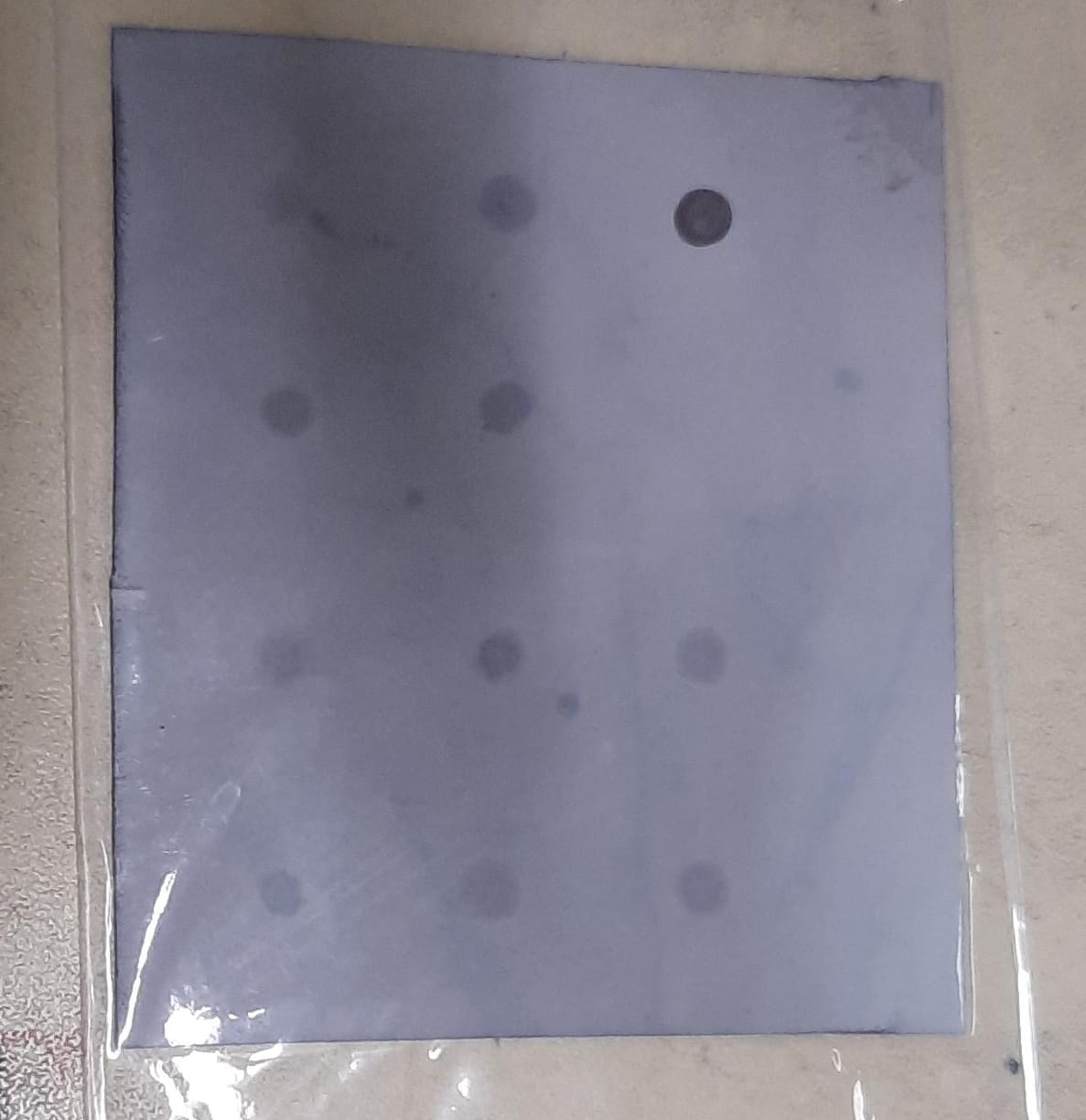

Supplement: Supplementary file 1 — Supplementary Information. [file 41598_2022_23130_MOESM1_ESM.docx]
